# Targeting zonulin and intestinal epithelial barrier function to prevent onset of arthritis

**DOI:** 10.1038/s41467-020-15831-7

**Published:** 2020-04-24

**Authors:** Narges Tajik, Michael Frech, Oscar Schulz, Fabian Schälter, Sébastien Lucas, Vugar Azizov, Kerstin Dürholz, Franziska Steffen, Yasunori Omata, Andreas Rings, Marko Bertog, Aroldo Rizzo, Aida Iljazovic, Marijana Basic, Arnd Kleyer, Stephan Culemann, Gerhard Krönke, Yubin Luo, Klaus Überla, Udo S. Gaipl, Benjamin Frey, Till Strowig, Kerstin Sarter, Stephan C. Bischoff, Stefan Wirtz, Juan D. Cañete, Francesco Ciccia, Georg Schett, Mario M. Zaiss

**Affiliations:** 1 Department of Internal Medicine 3, Rheumatology and Immunology, Friedrich-Alexander-University Erlangen-Nürnberg (FAU) and Universitätsklinikum Erlangen, Erlangen, Germany; 2 Deutsches Zentrum für Immuntherapie (DZI), Erlangen, Germany; 3 Department of Nutritional Medicine, University of Hohenheim, Stuttgart, Germany; 4 Institute of Cellular and Molecular Physiology, Friedrich-Alexander University Erlangen-Nürnberg, Erlangen, Germany; 5 Dipartimento Biomedico di Medicina Interna e Specialistica, University of Palermo, Palermo, Italy; 6 Helmholtz Centre for Infection Research, Braunschweig, Germany; 7 Institute for Laboratory Animal Science, Hannover Medical School, Hannover, Germany; 8 Department of Rheumatology & Immunology, West China Hospital, Chengdu, China; 9 Institute of Clinical and Molecular Virology, Friedrich-Alexander University Erlangen-Nürnberg, Erlangen, Germany; 10 Department of Radiation Oncology, Friedrich-Alexander-Universität Erlangen-Nürnberg, Universitätsklinikum Erlangen, Erlangen, Germany; 11 Department of Internal Medicine 1, University of Erlangen-Nürnberg, Erlangen, Germany; 12 Departamento de Reumatología, Hospital Clínic de Barcelona e IDIBAPS, Barcelona, Spain

**Keywords:** Autoimmunity, Rheumatoid arthritis

## Abstract

Gut microbial dysbiosis is associated with the development of autoimmune disease, but the mechanisms by which microbial dysbiosis affects the transition from asymptomatic autoimmunity to inflammatory disease are incompletely characterized. Here, we identify intestinal barrier integrity as an important checkpoint in translating autoimmunity to inflammation. Zonulin family peptide (zonulin), a potent regulator for intestinal tight junctions, is highly expressed in autoimmune mice and humans and can be used to predict transition from autoimmunity to inflammatory arthritis. Increased serum zonulin levels are accompanied by a leaky intestinal barrier, dysbiosis and inflammation. Restoration of the intestinal barrier in the pre-phase of arthritis using butyrate or a cannabinoid type 1 receptor agonist inhibits the development of arthritis. Moreover, treatment with the zonulin antagonist larazotide acetate, which specifically increases intestinal barrier integrity, effectively reduces arthritis onset. These data identify a preventive approach for the onset of autoimmune disease by specifically targeting impaired intestinal barrier function.

## Abstract (web summary)

Intestinal dysbiosis is associated with an ever-growing list of autoimmune diseases. Here the authors show that both mice and humans with autoimmune arthritis can have dysbiosis and barrier leakiness prior to major signs of inflammatory arthritis, and treatment of mice with a zonulin antagonist can limit collagen-induced arthritis.

## Introduction

Altered intestinal microbiota composition, termed “dysbiosis”, is associated with autoimmune diseases, particularly type 1 diabetes^1^, multiple sclerosis^2^, systemic lupus erythematosus^3^ and rheumatoid arthritis (RA)^4,5^. Several findings support the hypothesis that the onset of RA is linked to the intestinal microbiota, for example that cell wall fragments from various intestinal bacteria have been found to be arthritogenic^6,7^, some drugs used to treat arthritis have antimicrobial effects (chloroquine, sulfasalazine and minocycline)^8–10^ and the restoration of eubiosis in arthritis patients showing clinical improvement^7,11^. Moreover, diet as the main microbiota influencing factor has been shown to influence arthritis^12,13^. However, direct mechanistic links between the gut microbiota and onset of autoimmune diseases are unclear.

The permeability against small substances of the intestinal epithelium depends on the regulation of intercellular tight junctions (TJ). Zonula occludens toxin (Zot, or zonulin), an enterotoxin secreted by intestinal epithelial cells following stimuli from diets or microbiota, was shown to be a potent regulator of TJ competency and intestinal barrier function. Importantly, breakdown of the intestinal barrier, e.g. by apoptosis of intestinal epithelial cells in response to microbial infection, enables a proinflammatory environment including differentiation of autoreactive Th17 cells and other T helper cells^14^. Our data show that in mice intestinal barrier function is impaired before the clinical onset of arthritis, while in humans serum markers associated with impaired intestinal barrier function are also increased before the onset of RA and associated with a higher risk to develop RA later on. We identify zonulin family peptide (zonulin) as molecular factor that triggers the onset of arthritis by regulating intestinal barrier function. Zonulin reduces the expression of intestinal TJ proteins, induces T-cell-mediated mucosal inflammation and controls the transmigration of immune cells from the gut into the joints. Moreover, therapeutic restoration of intestinal barrier function, e.g. by specific targeting of zonulin, not only prevents the transmigration of immune cells from the gut to the joint but also partially protects from the onset of arthritis. These data unravel a mechanism that explains the transition from asymptomatic autoimmunity to inflammatory disease. Since early treatment of autoimmune diseases such as RA leads to better long-term outcomes and higher chances of drug-free remission^15^, the direct targeting of intestinal barrier function at onset of arthritis provides an opportunity to modulate the development of autoimmune diseases, i.e. the onset of inflammation in RA.

## Results

### Zonulin expression correlates with onset of rheumatoid arthritis

**Fig. 1:**
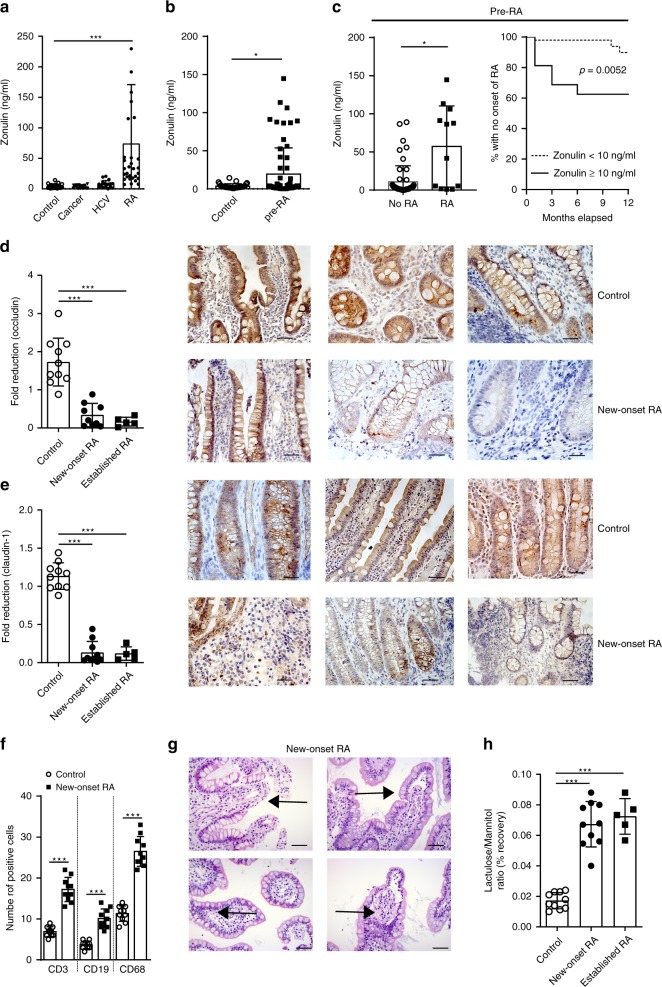
Zonulin family peptide levels and intestinal barrier dysfunction in human rheumatoid arthritis. **a** Serum zonulin family peptide (zonulin) levels in healthy controls (controls) (*n* = 41), cancer (*n* = 19), hepatitis C virus (HCV) (*n* = 21), and rheumatoid arthritis (RA) (*n* = 40) patients. **b** Serum zonulin levels in healthy controls (*n* = 41) and pre-RA patients (*n* = 32). **c** Serum zonulin levels in pre-RA patients with (*n* = 12) or without (*n* = 53) later development of RA (left panel); Kaplan–Meier graph showing loss of healthy state and progression to RA patients with low (<10) or elevated (≥10) zonulin levels over month relapsed (right panel). **d** Fold reduction of occludin expression determined in histological gut biopsy sections in healthy controls (*n* = 10), new-onset (*n* = 9) and established RA patients (*n* = 5) with representative microphotographs (occludin in brown) from three healthy control and three new-onset RA patients. Size bar: 20 µm. **e** Fold reduction of claudin-1 expression determined in histological gut biopsy sections in healthy controls (*n* = 10), new-onset (*n* = 10) and established RA patients (*n* = 5) with representative microphotographs (claudin-1 in brown) from three healthy control and three new-onset RA patients. Size bar: 20 µm. **f** Immunohistological quantification of gut infiltrating T cells (CD3), B cells (CD19) and macrophages (CD68) in gut biopsies from healthy controls or new-onset RA patients from stained gut biopsies (*n* = 10). **g** Representative H&E-stained gut biopsy microphotographs from a new-onset RA patient; arrows: signs of inflammation. Size bar: 20 µm. **h** Intestinal permeability assessed by lactulose/mannitol recovery ratio in urine of healthy controls (*n* = 10), and new-onset (*n* = 10) and established (*n* = 5) RA patients after 24 h. Data are expressed as the mean ± s.d. Statistical difference was determined by one-way ANOVA. *p < 0.05; ***p < 0.001. Source data are provided as a Source Data file.

The permeability and barrier function of the intestinal epithelium depends on the regulation of intercellular TJ. Among other factors, intestinal permeability is regulated by zonula occludens toxin (Zot, or zonulin)-mediated disengagement of the protein ZO-1 from the TJ protein complex^16^. Zonulin is secreted by intestinal epithelial cells following stimuli from diet or microbiota. We first assessed serum zonulin levels in two independent cohorts of established RA and found elevated zonulin levels compared to healthy controls (Fig. 1a, Supplementary Fig. 1a). Patients with chronic infections (hepatitis C infection; HCV) or cancer showed no elevation of zonulin (Fig. 1a). Furthermore, zonulin was already increased in two independent cohorts in a subset of patients with RA-specific autoimmunity that had not yet developed the disease (“pre-RA”) (Fig. 1b, Supplementary Fig. 1a). To add more evidence on intestinal barrier dysfunction, we also analyzed serum sCD14 levels, which has been described as marker of microbial translocation. Soluble CD14 levels were correlated with zonulin levels (Supplementary Fig. 1b), while no such correlation was found between the C-reactive protein (CRP) or the erythrocyte sedimentation rate (ESR) (Supplementary Fig. 1c). Longitudinal analysis revealed that pre-RA individuals with elevated zonulin levels (>10 ng/ml) had a high risk to develop RA within 1 year (Fig. 1c).

### Intestinal barrier changes at the onset of human RA

To further confirm intestinal barrier dysfunction during RA development, ileal mucosal biopsies from ten new-onset treatment-naïve RA and ten established active RA patients were collected (see also Supplementary Table 1). New-onset and established RA patients revealed lower expression of TJ proteins occludin and claudin-1 in intestinal epithelial cells than healthy controls on the RNA levels and in respective immune histology (Fig. 1d, e). No difference was observed between new-onset RA and established RA patients. Furthermore, the number of T cells (CD3), B cells (CD19) and macrophages (CD68) was increased in the lamina propria of new-onset treatment-naïve RA patients (Fig. 1f), which was associated with leukocyte infiltration in H&E-stained ileal biopsies (Fig. 1g). Functional assessment of intestinal permeability in these patients showed increased lactulose/mannitol recovery in the urine characteristic for impaired intestinal permeability (Fig. 1h). Taken together, these data point to a disruption of the intestinal epithelial barrier function early in the course of RA development.

### Intestinal inflammation precedes the onset of arthritis

**Fig. 2:**
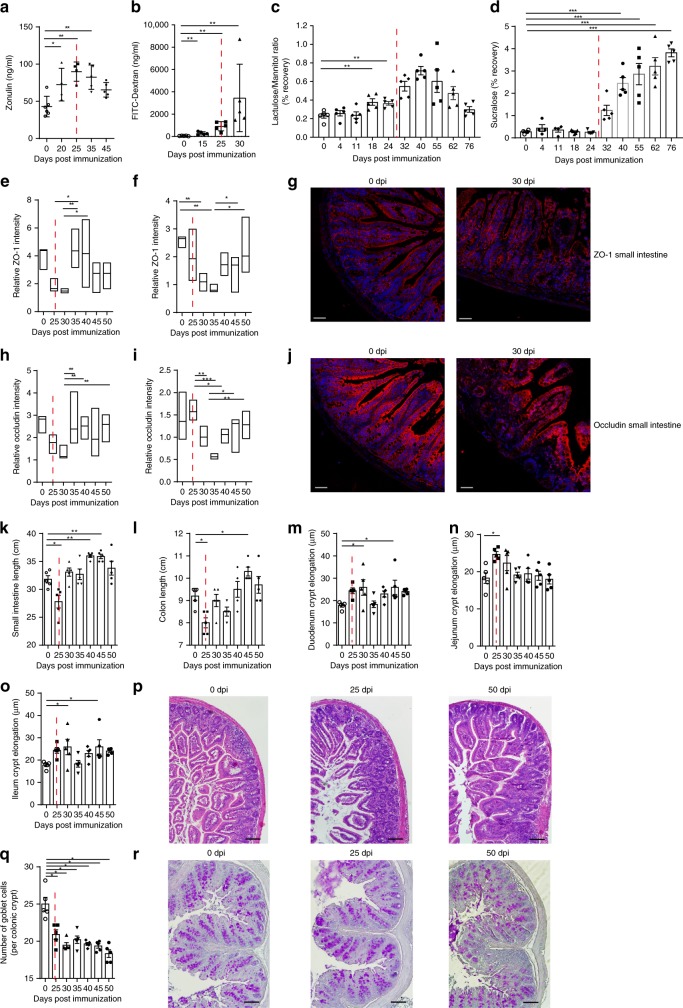
Zonulin upregulation, decrease in tight junction proteins and barrier dysfunction before onset of arthritis. **a** Time course of serum zonulin levels in mice induced for collagen-induced arthritis (CIA) (*n* = 5). **b**–**d** Time course of intestinal permeability assessed by **b** the serum FITC-Dextran, **c** lactulose/mannitol urine recovery ratio and **d** urine sucralose recovery ratio in mice induced for CIA (*n* = 5). **e**, **f** Quantification of ZO-1 (red) expression determined in histological sections of **e** the ileum (*n* = 6) and **f** the colon (*n* = 6) presented as floating bars (min to max) with line at median. **g** Representative microphotographs of ZO-1 (red) expression in small intestine. Size bar: 50 µm. **h**, **i** Quantification of occludin expression (red) in histological sections of **h** the ileum (*n* = 6) and **i** the colon (*n* = 6) presented as floating bars (min to max) with line at median with **j** representative microphotographs of occludin (red) expression in small intestine. Size bar: 50 µm. **k**, **l** Time course of the length measurement of **k** the small intestine and **l** the colon in mice induced for CIA. **m**–**o** Time course of **m** duodenal, **n** jejunal and **o** ileal crypt elongation with **p** representative H&E-stained sections from the ileum. Size bar: 200 µm. **q** Time course of goblet cell numbers in the colon and **r** representative PAS-stained sections from the colon. Size bar: 200 µm. Data are derived from two (**b**–**j**), three (**a**), or four independent (**k**–**r**) experiments and expressed as the mean ± s.d. Statistical difference was determined by one-way ANOVA. *p < 0.05; **p < 0.01; ***p < 0.001. Dashed red line: time point of clinical disease onset. Source data are provided as a Source Data file.

We next performed time-course analysis of serum zonulin levels in collagen-induced arthritis (CIA) of mice. Zonulin levels increased well before the onset of CIA 25 days post immunization (dpi) (Fig. 2a). In line with these observations, CIA mice showed increased small intestine permeability for FITC-dextran MW 4000 (FD4) at day 15 (Fig. 2b) and for lactulose/mannitol at day 18 post immunization (Fig. 2c), which was later followed by increased colon permeability at day 32, as measured by sucralose recovery in the urine (Fig. 2d). Furthermore, expression of TJ proteins ZO-1 (Fig. 2e–g) and occludin (Fig. 2h–j) was decreased before the onset of arthritis in the small intestine and shortly after onset of arthritis in the colon. Similar results were found at mRNA expression level with downregulation of ZO-1 and occludin before the onset of arthritis (Supplementary Fig. 2a, b), while the expression of ion and water channel-forming TJ proteins claudin-2 and -15 went up (Supplementary Fig. 2c, d). In addition, small intestine and colon length were transiently reduced at the onset of arthritis (Fig. 2k, l) along with crypt elongations in the small intestine (Fig. 2m–p) and reduced goblet cell numbers in the colon (Fig. 2q, r).

### T cells accumulate in the intestine before arthritis onset

To investigate cellular intestinal alterations that precede the onset of arthritis in more detail, we analyzed T-cell subsets in the intestine at different time points during the CIA time course. The pathogenesis of T-cell-mediated inflammation in the intestine and the joints has been described to be Th1 and Th17 cell-dependent^17,18^. Interestingly, we observed increased frequencies of Th1 (CD4^+^IFNγ^+^) (Supplementary Fig. 3a) and Th17 (CD4^+^IL-17^+^) cells (Supplementary Fig. 3b) in the small intestine and colon of CIA mice during the initiation phase of arthritis. In contrast, Th2 (CD4^+^IL-4^+^) and Treg cells (CD4^+^Foxp3^+^), known to play critical roles counteracting inflammation in arthritis^19,20^, were increased only in the late stages of arthritis (Supplementary Fig. 3c, d)^19,21^. Detailed gating strategies are shown in Supplementary Fig. 3e, f. Collectively, histological and cellular signs of intestinal inflammation precede the onset of arthritis and may trigger the initiation of the disease. Analysis of the synovial tissue and popliteal lymph nodes (pLN) by flow cytometry analysis at this early time points (25 days after immunization) did not reveal any changes in the total synovial CD4^+^ T-cell or B220^+^ B-cell frequencies (Supplementary Fig. 4b), as well as no changes in pLN Th1 (CD4^+^IFNγ^+^) and Th2 (CD4^+^IL-4^+^) frequencies, while Th17 (CD4^+^IL-17^+^) cell frequencies were increased (Supplementary Fig. 4c).

### Intestinal microbial changes in the pre-phase of arthritis

Among several potential stimuli that can trigger zonulin release, intestinal microbiota composition was identified as being one of the most potent^16^. Microbial dysbiosis is known to affect intestinal short chain fatty acid (SCFA) levels^22,23^. The SCFA butyrate has been shown to decrease bacterial translocation and strengthen intestinal barrier function^24^. CIA mice showed a change in the intestinal microbiota genera and families before the onset of arthritis at day 20 after immunization (Supplementary Fig. 5a–d). Moreover, such microbiota changes occurred already very early in the disease process finding differences in the small intestinal fecal content as early as day 3 after immunization, including an enrichment of Lactobacillaceae_OTU3 and decreased Lactobacillaceae_OTU1 or Bacteroidales S24_7_OTU9 (Supplementary Fig. 5e). PCoA plot analyses showed that these changes are mainly attributed to the day post immunization (Supplementary Fig. 5f). In accordance with these changes in the microbiota composition after CIA induction, butyrate levels changed, dropping just before the onset of arthritis indicative for a changed microbiota composition and decreased function of the intestinal barrier^25^ (Supplementary Fig. 5g).

### Breach in intestinal permeability is mediated by the microbiota

**Fig. 3:**
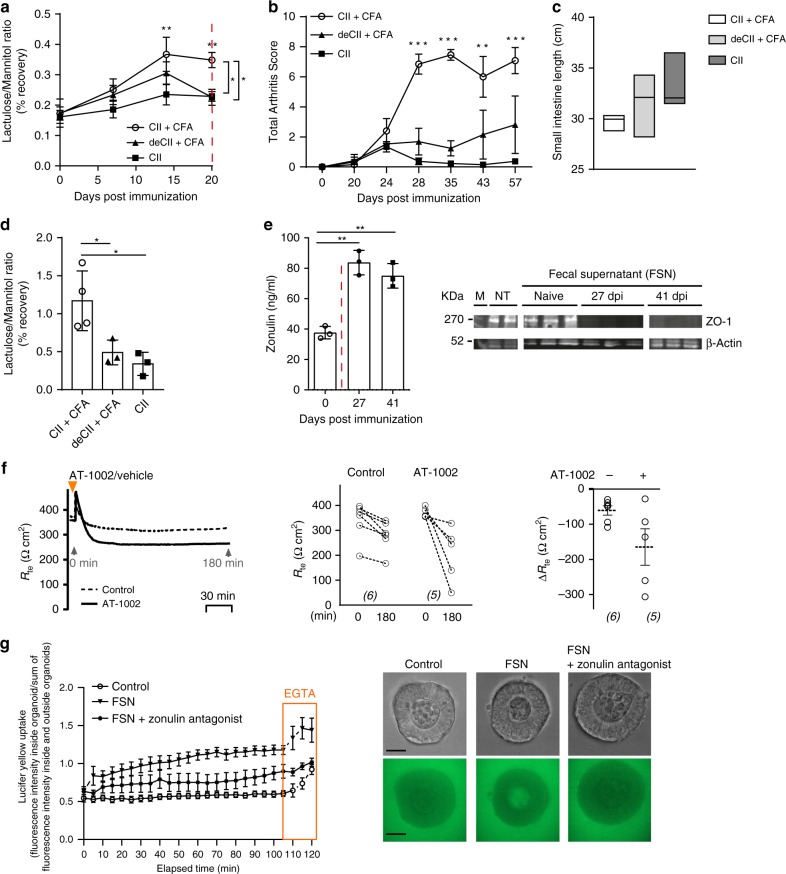
Dysbiotic microbiota from arthritis mice transfers leaky barrier and mucosal inflammation to GF mice. **a** Intestinal permeability assessed by the lactulose/mannitol urine recovery ratio in mice using different collagen-induced arthritis (CIA) immunization protocols namely collagen type II + CFA (CII + CFA), denaturated collagen type II + CFA (deCII + CFA) or collagen type II only (CII) (*n* = 5). **b** Arthritis scores in mice induced for CIA using different immunization protocols (*n* = 5). **c** Length measurement of the intestine at 20 days post immunization (dpi) in CIA mice (*n* = 4) presented as floating bars (min to max) with line at median. **d** Intestinal permeability assessed by the lactulose/mannitol urine recovery ratio in germ-free (GF) mice reconstituted with the donor microbiota from mice immunized with different CIA immunization protocols at 20 dpi (*n* = 4). **e** Time course of serum zonulin levels and western blot analysis of ZO-1 expression in Caco-2 cells after 24 h stimulation with intestinal fecal supernatants (FSN) (*n* = 3). **f** Transepithelial electrical resistance (TEER) changes in mice induced for CIA treated with the zonulin agonist (AT-1002) (*n* = 6). **g** Intestinal organoids from wild-type mice treated with fecal supernatant (FSN) from mice with collagen-induced arthritis at 35 dpi, FSN and zonulin antagonist, or PBS (control). Upon addition of Lucifer yellow (457 Da), confocal fluorescent images were captured every 5 min for 120 min. At 105 min, EGTA was added as a positive control for the ability of the organoids to take up Lucifer yellow. Fluorescence was determined in the organoid lumen and outside of the organoid. Relative intensity values were calculated (fluorescence inside/outside) and are shown for each time point. Each point represents mean values, measured in ten organoids derived from two independent experiments (*n* = 10). Representative images at time point 55 min are shown. Upper panel: brightfield; lower panel: Lucifer yellow (green), size bar: 50 μm. Data are derived from two (**d**–**g**) or three (**a**–**c**) independent experiments and expressed as the mean ± s.d. Statistical difference was determined by one-way (**d**, **e**) or two-way ANOVA (**a**, **b**, **g**). *p < 0.05; **p < 0.01; ***p < 0.001. Dashed red line: time point of clinical disease onset. Source data are provided as a Source Data file.

To further examine the link between the arthritis-related immune response, changed microbiota and intestinal epithelial barrier dysfunction, we applied different immunization protocols including (i) “arthritogenic” collagen type II + complete Freunds adjuvans (CII + CFA), (ii) denaturized CII + CFA (deCII + CFA) and (iii) CII without CFA (CII). We observed that barrier disruption only occurred in mice receiving the “arthritogenic” immunization and started as early as 7 days after immunization (Fig. 3a). Hence, arthritis only developed in mice, which developed increased intestinal permeability (based on lactulose/mannitol ratio measurements) and intestinal inflammation (based on small intestine length measurement) (Fig. 3b, c). Furthermore, only fecal ileal samples from mice immunized with CII + CFA, but not from the other regimen increased gut permeability (based on lactulose/mannitol ratio measurements) (Fig. 3d) when transferred into germ-free (GF) mice.

Moreover, in vitro stimulation of the intestinal epithelial Caco-2 cell line with fecal supernatants (FSN) of mice induced for CIA, showing elevated serum zonulin levels, effectively suppressed ZO-1 protein expression (Fig. 3e). Overall, these data support the human observations and show increased zonulin levels, reduced TJ protein complex expression and impaired intestinal barrier function preceding the onset of arthritis. As previously shown^26^, recombinant AT-1002, the active domain of *Vibrio cholerae’s* zonula occludens toxin (ZOT, zonulin) on average tended to decrease the transepithelial electrical resistance (TEER) in Caco-2 cell monolayers, indicating the permeability of epithelial cell monolayers (Fig. 3f). Hence, we tested whether blockade of zonulin overcomes the induction of epithelial permeability by FSN from mice induced for CIA. Lucifer yellow staining of intestinal organoids showed that FSN from mice induced for CIA enhanced permeability, which was reversed when zonulin was blocked by larazotide acetate (Fig. 3g), suggesting the microbial dysbiosis in mice induced for CIA impairs intestinal barrier via zonulin.

### Reducing intestinal barrier permeability attenuates arthritis

**Fig. 4:**
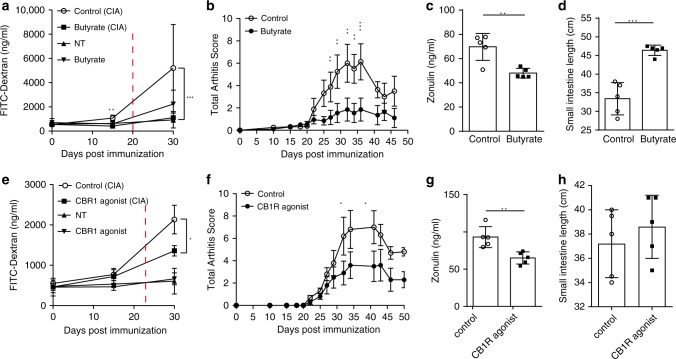
Targeting intestinal barrier dysfunction before arthritis onset attenuates development of arthritis. **a**, **b** Time course of **a** intestinal barrier permeability (*n* = 5) and **b** total arthritis scores in collagen-induced arthritis (CIA) or nontreated healthy (NT) mice with or without butyrate treatment (*n* = 5). **c**, **d** Effect of butyrate treatment of mice induced for CIA on their **c** serum zonulin levels (*n* = 5) and **d** intestinal length measurements (*n* = 5). **e**, **f** Time course of **e** intestinal barrier permeability (*n* = 5) and **f** total arthritis scores in CIA or nontreated healthy mice with or without CB1R treatment (*n* = 5). **g**, **h** Effect of CB1R agonist treatment of mice induced for CIA on **g** serum zonulin levels (*n* = 5) and **h** intestine length (*n* = 5). Data are derived from two (**e**–**h**) or four (**a**–**d**) independent experiments and expressed as the mean ± s.d. Statistical difference was determined by Students’ *t* test, two-tailed (**c**, **d**, **g**) or two-way ANOVA (**a**, **b**, **e**, **f**). *p < 0.05; **p < 0.01; ***p < 0.001. Dashed red line: time point of clinical disease onset. Source data are provided as a Source Data file.

Since our results suggested that increased zonulin levels along with impaired intestinal permeability for lactulose and FITC-Dextran (FD4) are preceding the onset of murine and human arthritis, we sought an intervention that strengthens the intestinal barrier and may disrupt the transition from autoimmunity to inflammation and thereby inhibit the onset of arthritis. Therefore, we first strengthened intestinal barrier function by treatment with butyrate as it was described to have beneficial effects on gut permeability^25^. Butyrate treatment in the drinking water from day 0 post immunization prevented the early increase in intestinal permeability for FITC-Dextran (FD4) at day 15 after immunization (Fig. 4a). In addition, butyrate treatment attenuated CIA onset (Fig. 4b), decreased serum zonulin concentrations (Fig. 4c), and reduced inflammation-mediated small intestinal shortening (Fig. 4d). Moreover, butyrate treatment improved intestinal barrier function by modulating TJ protein mRNA expression levels (Supplementary Fig. 6a). Given that the intestinal epithelial barrier function was found to be positively regulated by activation of the intestinal cannabinoid type 1 receptor (CB1R)^27^, we treated mice with ACEA (arachidonyl-2-chloroethylamide), a selective CB1R agonist^28^ during the autoimmune noninflammatory pre-phase of CIA from day 17 to day 27 after immunization. ACEA treatment led to reduced intestinal barrier permeability for FITC-Dextran (FD4) (Fig. 4e), attenuated clinical signs of arthritis (Fig. 4f) along with a reduction in serum zonulin levels (Fig. 4g) as well as no morphological signs of gut inflammation (Fig. 4h).

### Targeting zonulin attenuates arthritis

**Fig. 5:**
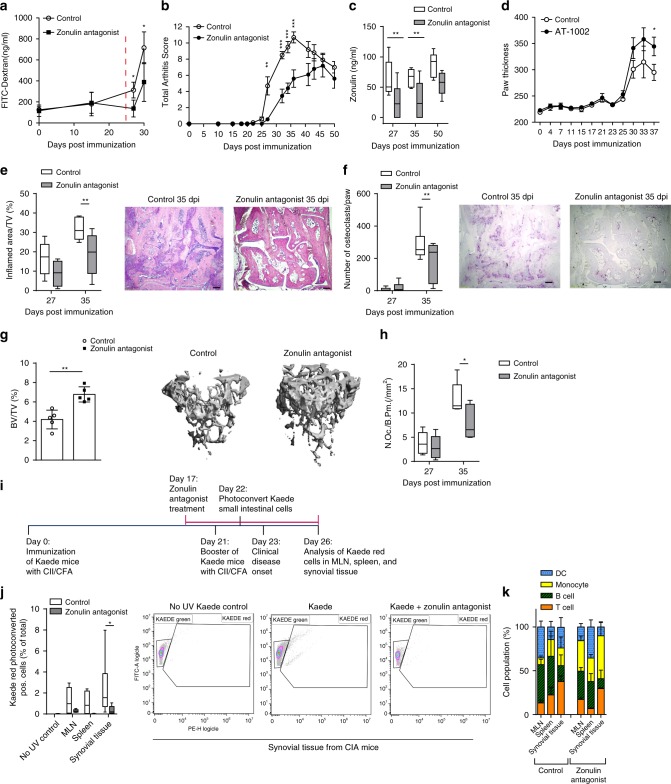
Short-term zonulin antagonist treatment improves bone homeostasis. **a**–**c** Time course of **a** intestinal barrier permeability (*n* = 5), **b** total arthritis scores (*n* = 5) and **c** serum zonulin levels in collagen-induced arthritis (CIA) mice treated with Larazotide (zonulin antagonist) between 17 and 27 days post immunization (dpi) (*n* = 5, box and whiskers showing 1–99 percentile). **d** Time course of arthritis scores in mice induced for CIA treated with the zonulin agonist (AT-1002) (*n* = 5). **e**, **f** Histological analysis of tarsal joints after zonulin antagonist treatment showing **e** inflamed area and respective H&E-stained sections (size bar: 500 µm) (*n* = 5, showing 1–99 percentile) as well as **f** osteoclast numbers with respective TRAP-stained sections (size bar: 500 µm) (*n* = 5, showing 1–99 percentile). **g** Bone densities in mice treated with zonulin antagonist and respective micro-CT images (*n* = 5). **h** Osteoclast numbers per bone parameter in the tibia of CIA mice treated with zonulin antagonist between 17 and 27 days post immunization (*n* = 5, showing 1–99 percentile). **i** Schematic overview of cell trafficking experiments using photoconvertible Kaede mice. **j** Quantification of photoconverted cells from Kaede mice in lymphoid organs and the synovial tissue following short-term zonulin antagonist treatment (*n* = 8). **k** Cell populations identified in MLN, spleen and synovial tissue from Kaede CIA mice at day 26 post immunization (*n* = 6). Data are derived from three independent experiments and expressed as the mean ± s.d. Statistical difference was determined by Students’ *t* test, two-tailed (**g**) or two-way ANOVA (**a**–**f**, **h**, **i**). *p < 0.05; **p < 0.01; ***p < 0.001. Dashed red line: time point of clinical disease onset. Source data are provided as a Source Data file.

Next, and most importantly, we directly blocked zonulin by larazotide acetate (AT-1001), a first-in-class TJ regulator, currently used in phase III clinical trials for celiac disease^29^. This octapeptide is related to the originally identified ZOT produced by *V. cholera*. It acts locally to decrease tight junction permeability by blocking zonulin receptors and promoting TJ assembly and actin filament rearrangement thus preventing actin rearrangement in response to gluten or microbial stimuli^30,31^. Ten days oral treatment of CIA mice with the zonulin antagonist larazotide shortly before the onset of arthritis prevented the observed increase in intestinal barrier permeability for FITC-Dextran (FD4) and attenuated arthritis symptoms (Fig. 5a, b). Moreover, zonulin antagonist treatment 17–27 dpi lead to a sustained decrease in serum zonulin levels (Fig. 5c) and positively modulated tight junction mRNA expression in the ileum (Supplementary Fig. 6b). Conversely, further promoting zonulin action by short-term treatment with the agonist peptide AT-1002 led to an exacerbation of arthritis (Fig. 5d). Histologic evaluation of the joints revealed less severe synovitis (Fig. 5e), lower numbers of osteoclasts (Fig. 5f) and better preservation of bone (Fig. 5g), including a reduction of osteoclast-covered bone area (Fig. 5h) in mice receiving zonulin blockade by larazotide. These data indicate that specific restoration of intestinal epithelial integrity by zonulin blockade affects the development of arthritis.

### Lamina propria lymphocytes migrate to synovial tissues

Next, we investigated the mechanism by which maintenance of intestinal barrier function inhibits arthritis onset. Indeed, short-term treatment with zonulin antagonist larazotide mitigated intestinal inflammation with abrogation of small intestine and colon shortening (Supplementary Fig. 7a). Also, T-cell homeostasis in the spleen and the intestine were shifted towards an anti-inflammatory proresolving phenotype by zonulin blockade during the initiation phase of CIA (Supplementary Fig. 7b, c). Hence, in the spleens CD4^+^FoxP3^+^ Tregs were increased, while proarthritic Th1 (CD4^+^Tbet^+^) and Th17 (CD4^+^RORγt^+^) were decreased (Supplementary Fig. 7b). In the lamina propria, Th1 cells were also decreased upon zonulin blockade, while CD4^+^Gata3^+^ Th2 cells were increased (Supplementary Fig. 7c). Of note, levels of CII-specific IgG, IgG2a and IgG2b were not changed by the zonulin antagonist (Supplementary Fig. 8a–c).

To investigate the migration of T cells from the small intestine into the joints, we used mice engineered to ubiquitously express Kaede^32^. Kaede is a photoconvertible protein, which permanently changes its fluorescence emission from green (518 nm) to red (582 nm) upon photoactivation with near-UV light. After selective exposure of the small intestine, Kaede-photo-conversion was found to be specific to cells in the small intestine. As shown in the schematic overview of the experimental set-up (Fig. 5i), small intestinal cells in CIA Kaede mice were photoconverted before the onset of clinical arthritis at day 22 after immunization and tissues were analyzed 4 days later by flow cytometry. At day 26 after immunization, CIA Kaede mice showed migrated photoconverted Kaede red^+^ cells originating from the small intestine in the lymph nodes, the spleen and most prominent in the synovial tissue of the joints (Fig. 5j). Interestingly, the percentage of Kaede red^+^ CD4^+^ T cells was highest in the synovial tissues (Fig. 5k), demonstrating the preferential migration of T cells from the small intestine into the joints. Most importantly, this cellular trafficking of gut-primed immune cells to secondary lymphoid organs and the synovial tissues was prevented by short-term zonulin antagonist treatment (Fig. 5j, k). In summary, these data show the anzonulin-dependent migration of immune cells from the intestine to the joints during the onset of arthritis.

## Discussion

Here we show that zonula occludens toxin (Zot, or zonulin), a potent regulator for intestinal TJ competency and intestinal barrier function, is controlling the onset of murine and human arthritis. Changes in the intestinal barrier function have been reported in the clinical phase of inflammatory bowel disease but also in spondyloarthritis and type 1 diabetes mellitus^33–35^. Herein, we demonstrate that the disruption of the intestinal barrier function occurs before the onset of the inflammatory phase of autoimmune murine and human arthritis identifying it as checkpoint for the transition from autoimmunity to inflammation. We show that zonulin is a central factor in this process as it disrupts intestinal TJ proteins, enhances intestinal permeability and leads to Th1 and Th17 infiltration in the lamina propria before the onset of murine and human arthritis. While ZO-1, occludin and claudin-1 expression was reduced, expression of paracellular cation- and water- channel-forming claudins 2 and 15 was increased. Increased claudin-2 and claudin-15 mRNA levels (both forming paracellular cation and water channels) may explain the observed changes in in vitro TEER measurements following AT-1002 treatment of Caco-2 cell monolayers. In addition, in vivo, decreased occludin levels in combination with increased claudin-2 and claudin-15^36^ might in synergy result in the observed increased barrier leakiness in CIA mice. ZO-1 is an intracellular located adaptor protein that binds numerous transmembrane and cytoplasmic proteins and is required for assembly of both adherens and TJ. TJ paracellular barrier function is based on two independently regulated routes^37^, namely the pore pathway: a highly selective route for the flux of ions and water, that is regulated by claudins. This route could be experimentally measured by the assessment of TEER. While on the other hand there exist the leak pathway that regulates the passage of larger molecules and is measured by the passage of fluorescent tracers (FITC) across epithelial monolayers. These two routes can be regulated independently^37^. For example, depletion of ZO-1 destabilizes the barrier to large solutes, but does not disrupt the claudin-based pores^38^. Currently, the exact contribution of occludin in contrast to ZO-1 to the barrier function is debated and requires further research. Here, we could show that a zonulin-dependent transmigration of immune cells from the gut into the joints occurs during the onset of arthritis.

Our data also show that zonulin-dependent increase of intestinal permeability essentially results from microbial dysbiosis initiated by the arthritogenic combination of auto-antigen and adjuvant. This finding is in accordance with a previous study showing that adjuvant alone was not sufficient to initiate mucosal inflammation^39^. In support of the role of microbial dysbiosis, stool transfer was sufficient to initiate intestinal barrier changes in germ-free mice. Furthermore, functional experiments on intestinal epithelial cells showed that the permeability of intestinal organoids is increased by the feces of CIA mice in a zonulin-dependent manner. Recombinant zonulin was proven to increase the intestinal permeability ex vivo^40^ and in vitro^16^. In both settings this increase could be blocked by cleaving zonulin or treatment with a zonulin binding inhibitor. Moreover, as shown by the same group, recombinant zonulin-induced effects were completely reversible following its withdrawal in Rhesus monkey intestinal ileal tissues using Ussing chamber experiments and and the effects could specifically be blocked by preincubation of zonulin with anti-Zot antibodies^41^. Together, these data highlight the potential of zonulin, more specifically of its active domain which is shared with Zot^42^, to increase intestinal permeability. Here, we could demonstrate that blocking zonulin with the specific antagonist larazotide acetate was sufficient to restore the impaired barrier function in intestinal organoid cultures exposed to complete fecal supernatants from CIA mice containing high zonulin levels. This finding is of direct physiological relevance, as all other active metabolites in the used fecal supernatants were still present but mere inhibition of zonulin was sufficient to restore the impaired intestinal barrier function.

Data from clinical observations and preclinical animal models suggest a step-wise process leading to autoimmune RA^17^. Genetic factors in conjunction with environmental triggers lead to breach of immune tolerance and a phase of clinically silent autoimmunity that can last for years. Later, in response to yet unidentified triggers, silent autoimmunity eventually turns into a clinically apparent inflammatory disease such as RA. Interfering with such triggers would allow retarding or even inhibiting the onset of the disease. Notably, currently available therapies for RA, such as cytokine (TNF and IL-6) blockers or inhibitors of the JAK-STAT pathway^43^, exclusively tackle the inflammatory phase of the disease but do not interfere with the underlying processes that initiate the onset of arthritis and therefore often need to be given life-long^44^.

We provide several lines of evidence that treatments that improve intestinal barrier function attenuate arthritic symptoms. Microbial metabolites such as butyrate, for instance, have shown to be potent regulators of zonulin and the intestinal barrier and appear to be essential mediators between microbial dysbiosis and barrier function. In accordance, treatment with butyrate not only restored epithelial barrier function and gastrointestinal permeability for FITC-Dextran (FD4) but also effectively inhibited arthritis. Even more specifically, specific zonulin inhibition during the preclinical phase of arthritis by larazotide reduced the later development of arthritis by nearly 50%. Larazotide treatment attenuated the enhanced intestinal permeability and blocked the migration of immune cells from the intestine to the joints (graphical summary in Supplementary Fig. 9). The implication of these findings is substantial, as it gives the chance to tackle the transition from autoimmunity to inflammation by restoring intestinal barrier function and thereby preventing the onset of RA. Such an approach also appears within short reach, as the zonulin antagonist larazotide is already used in phase III clinical trial for the treatment of celiac disease and thus represents an accessible target molecule to accomplish this task. Furthermore, elevated zonulin levels appear to be an interesting biomarker to identify those patients that are of highest risk to progress to RA, thus allowing a precision-medicine approach to target such individuals. In summary, these data support a gut-joint axis in RA, which is based on zonulin-mediated impairment of intestinal barrier function and which is drugable by the zonulin antagonist larazotide.

## Methods

### Study design

The objective of this study was to identify the effect of the TJ protein zonulin in CIA mice and RA patients as a mechanism contributing to the onset of clinical symptoms in arthritis. We hypothesized that preventing zonulin binding to intestinal epithelia cells decreased the here reported higher intestinal barrier permeability for FITC-Dextran (FD4) along with infiltration of proinflammatory cells in the lamina propria, leading to the prevention of arthritis. Using mice, human serum, and in vitro experiments, we analyzed the effect of short-term larazotide, a zonulin antagonist, treatment during preclinical phases of arthritis to show the existence of the gut−joint axis and open therapeutic opportunities to prevent arthritis. Except for 16S rRNA sequencing, all in vitro studies were based on at least three repeats and in vivo data on 5–10 mice per group with at least two independent repeats to achieve significant differences.

### Human participants

Serum zonulin levels were analyzed by ELISA (Cusabio Technology LLC, USA) and compared in cross-sectional manner in two cohorts: (i) 41 healthy female controls, 65 female predisease patients and 40 female RA patients from the Department of Internal Medicine 3 of the University of Erlangen-Nürnberg/Germany as well as (ii) 11 healthy female controls, 21 female predisease patients and 28 female RA patients from the Department of Rheumatology & Immunology of the West China Hospital in Chengdu/China. Healthy controls had to have (1) no presence or history of chronic joint pain/swelling, (2) no presence of systemic diseases and (3) no positivity for ACPA or rheumatoid factor (RF). Predisease individuals were defined as having RA-specific autoimmunity (ACPA assessed by testing positive for anticyclic citrullinated peptide 2 IgG autoantibody reactivity) but no past or present evidence of joint swelling according to phases C and D of the RA-at risk definition^45^. In longitudinal, analyzed pre-RA patients of Erlangen were followed for the later development of RA. Diagnosis of RA was based on arthritis fulfilling the ACR/EULAR 2010 criteria (38). Disease activity of RA was assessed by disease activity score 28 (DAS28). In the Erlangen cohort, 17/40 (42.5%) RA patients received biological disease modifying antirheumatic drugs (bDMARDs), mostly TNF inhibitors. Interestingly, in patients treated with bDMARDs, zonulin levels were lower than in those receiving conventional treatments (*p* = 0.05). The three groups (healthy controls, pre-RA and RA patients) were balanced for age, sex and body mass index within their respective cohorts. None of these participants had present or past symptoms of a chronic inflammatory bowel disease or celiac disease. Participants were negative for HLA27 and none had clinical signs of spondyloarthritis or psoriasis. Baseline zonulin levels were compared between the groups as well as in pre-RA patients, who developed RA and those who did not develop RA (Erlangen cohort). In addition, serum samples from patients with chronic infections (hepatitis C virus, HCV; *N* = 21) and cancer (*N* = 19, NCT02600065 and NCT03453892) were analyzed for serum zonulin levels. Written informed consent was obtained from all participants. All analyses were approved by the institutional review board of the University Clinic of Erlangen (#4564) and the West China Hospital of the Sichuan University (ChiCTR1900022605).

In addition, serial ileal mucosal biopsies from ten healthy controls (undergoing ileo-colonoscopy for screening purpose), ten consecutive new-onset treatment-naïve RA patients (eight females, two males) and five patients with active (DAS28 > 3.2) established RA were analyzed. These procedures were approved by the institutional review board of the Azienda Ospedaliera Universitaria Paolo Giaccone of the University of Palermo and patients provided written informed consents. Patients were balanced for age, sex and body mass index. RA patients fulfilled the ACR-EULAR 2010 criteria (38) and had to be positive for anti-CCP2 antibodies. Also, none of these patients had present or past symptoms of a chronic inflammatory bowel disease or celiac disease. Patients had to be negative for HLA27 and clinical signs of spondyloarthritis or psoriasis. Paired paraffin-embedded tissue and RNA samples were prepared from all patients to allow cross-referencing between histological assessments and qPCR gene expression analysis. Baseline characteristics of the patients are shown in Supplementary Table 1.

### Histology and immunohistochemistry in humans

Paraffin-embedded ileal samples were cut into 4-μm sections and stained with hematoxylin and eosin (H&E). Immunohistochemical analysis for CD3, CD19, CD68, occludin and claudin-1 was performed on 5-µm-thick paraffin-embedded sections from these biopsies and from tonsils (used as positive controls) as previously described^46^. Briefly, following rehydration, antigen was unmasked for 45 min at 95 °C using Dako Target retrieval solution (pH 6; Dako, Carpinteria, CA). Endogenous peroxidase was blocked for 10 min with Dako peroxidase blocking reagent, and nonspecific binding was blocked for 20 min with Dako protein block. The primary antibodies, mouse monoclonal anti-human CD3 (Leica Biosystems, dilution 1:100), CD19 (Dako, dilution 1:50), CD68 (Dako, dilution 1:100), occludin (Santa Cruz Biotechnology, dilution 1:100) and claudin-1 (Santa Cruz Biotechnology, dilution 1:100), were added and incubated for 1 h at room temperature. An isotype-matched irrelevant antibody was used as a negative control. Following three washes with Tris-buffered saline, slides were incubated for 30 min with peroxidase-conjugated Dako EnVision polymer. After three further washes, peroxidase activity was visualized using diaminobenzidine chromogen (Dako), and slides were lightly counterstained with hematoxylin before dehydration and mounting in DePex (VWR International, Oslo, Norway). The number of immune-reactive cells was determined by counting positively stained cells on photomicrographs obtained from three random high-power microscopic fields (×400 magnification) under a Leica DM2000 optical microscope, using a Leica DFC320 digital camera (Leica, Rijswijk, the Netherlands). AR and FC evaluated the ileal samples with no access to clinical data. The intra-rater agreement and the inter-rater agreement calculated by the Cohen’s K coefficient for the two observers were 0.88 and 0.78, respectively.

### RT-PCR in humans

Soon after removal, ileal biopsies were stored in RNAlater® solution (Applied Biosystems, Foster City, CA, USA). RT-PCR was performed as previously described^46^. Master mix and Taqman® gene expression assays for glyceraldehyde 3-phosphate dehydrogenase (GAPDH control) and target genes occludin (Hs05465837_g1) and claudin-1(Hs00221623_m1) were obtained from Applied Biosystems (Foster City, CA, USA). Data were quantified using sds 2.1 software and normalized using GAPDH as endogenous control. Relative changes in gene expression between HCs and RA samples were determined using the ΔΔCt method. Levels of the target transcript were normalized to a GAPDH endogenous control, constantly expressed in both groups (ΔCt). For ΔΔCt values, additional subtractions were performed between RA (*n* = 10) and HCs (*n* = 10) ΔCt values. Final values were expressed as fold of induction.

### RT-PCR in mice

Tissues were stored in RnaLater (Ambion) or directly transferred to TRIzol (Invitrogen). RNA was extracted according to the manufacturer’s instructions. Gene expression results are expressed as arbitrary units relative to expression of the house keeping gene GAPDH. Primer sequences are as follows: GAPDH: 5′-GGG TGT GAA CCA CGA GAA AT-3′ and 5′-CCT TCC ACA ATG CCA AAG TT-3′; ZO-1: 5′-CCA CCT CTG TCC AGC TCT TC-3′ and 5′-CAC CGG AGT GAT GGT TTT CT-3′; occludin: 5′-CCT CCA ATG GCA AAG TGA AT-3′ and 5′-CTC CCC ACC TGT CGT GTA GT-3′; claudin-1: 5′-TCC TTG CTG AAT CTG AAC A-3′ and 5′-AGC CAT CCA CAT CTT CTG-3′; claudin-2: 5′-TAT GTT GGT GCC AGC ATT GT-3′ and 5′-TCA TGC CCA CCA CAG AGA TA-3′; claudin-15: 5′-GCT TCT TCA TGT CAG CCC TG-3′ and 5′-TTC TTG GAG AGA TCC ATG TTG C-3′.

### Lactulose to mannitol ratio in humans

The ratio of urinary excretion of lactulose to mannitol (LA/MA) was used to measure the intestinal mucosal permeability in ten RA patients and ten healthy controls who underwent colonoscopy, with higher ratios indicative of increased intestinal permeability as previously described^33^.

### Animals

All mice were maintained under specific pathogen-free conditions at the Präklinisches Experimentelles Tierzentrum (PETZ), Erlangen, Germany and approved by the local ethics authorities of the Regierung of Unterfranken (#55.2-2532-2-424 and #55.2-2532-2-630). For all experiments, if not otherwise stated, DBA1/J female mice (8 weeks old) were used and purchased from Janvier Labs. Kaede mice were kindly provided by M. Tomura from the RIKEN Institute, Tokyo, Japan. Germ-free (GF) mice were kindly provided by M. Basic from the Hannover Medical School, Hannover, Germany. The animals were kept in the Franz-Penzoldt-Zentrum (FPZ) of the University Hospital Erlangen or the animal laboratory of Med3 under standardized husbandry conditions and hygiene management in accordance with Federation of European Laboratory Animal Science Associations (FELASA). The animals received water and feed ad libitum. The keeping rooms had a temperature of 22–23 °C and a humidity of 50–60%. There was also a 12-h light–dark rhythm in the holding rooms. Animals were kept in type II long cages, with maximum five animals.

### Mice studies

For all experiments, mice were acclimated for 1 week, followed by a 2-week co-housing period before the experiments started. Supplementation of butyrate (all Sigma-Aldrich, Germany) was done in the drinking water at a final concentration of 150 mM and changed every 3 days and control mice received pH and sodium-matched water. Zonulin antagonist (known as Larazotide acetate, or AT-1001, or INN-202) was purchased from BOC Sciences, NY, USA or later synthesized by GENAXXON biosciences, Ulm, Germany and effectiveness was compared in one side-by-side experiment using both purchased zonulin antagonist sources (BOC Sciences, NY, USA vs. GENAXXON biosciences) to confirm their biological function. Mice received 0.15 mg/ml zonulin antagonist in the drinking water and changed every day for 10 consecutive days between 17 and 27 dpi in the CIA model. For CAIA mouse model, C57BL/6 mice received 50 µg/mouse i.v. zonulin antagonist or vehicle (3% DMSO in phosphate‐buffered saline (PBS)) 1 day after CAIA induction. CB1 receptor agonist (ACEA; arachidonyl-2-chloroethylamide, Sigma-Aldrich, Germany) treatment was i.p. injections daily with 250 µg/mouse between 17 and 27 days post CII immunization. CIA was induced in 8-week-old female Kaede (C57BL/6J) or wild-type DBA/1J mice by subcutaneous injection at the base of the tail with 100 μl with 0.25 mg chicken type II collagen (Chondrex, Redmond, WA) in complete Freund adjuvant (Difco Laboratory, Detroit, MI), containing 5 mg/ml killed *Mycobacterium tuberculosis* (H37Ra). Mice were challenged after 21 days by intradermal immunization in the base of the tail with this emulsion. The paws were evaluated for joint swelling and grip strength three times per week. Each paw was individually scored using a 4-point scale: 0, normal paw; 1, minimal swelling or redness; 2, redness and swelling involving the entire forepaw; 3, redness and swelling involving the entire limp; 4, joint deformity or ankylosis or both. In addition, grip strength of each paw was analyzed on a wire 3 mm in diameter, using a score from 0 to −4 (0, normal grip strength; −1, mildly reduced grip strength; −2, moderately reduced grip strength; −3, severely reduced grip strength; −4, no grip strength at all). For photoconversion of Kaede-transgenic mice, the small intestine of anesthetized Kaede-transgenic mice was subjected to lighting using a BlueWave LED Prime UVA (Dymax).

### In vivo intestinal permeability measurements

Once every week, mice were housed in metabolic cages after a 4 h fasting of food and water and immediately after a gavage of 0.2 ml of a 10 ml sugar probe containing 100 mg of sucrose, 12 mg of lactulose, 8 mg of mannitol and 6 mg of sucralose. After the collection of urine the animals were placed in their respective cages, and provided with food and water. For the FITC-Dextran measurements, after 4 h fasting of food and water, mice were immediately orally gavaged with 200 μl of FITC-dextran (FD4) (440 mg/kg body weight), and blood was collected 4 h later. The concentration of the FITC-dextran was determined using a fluorimeter with an excitation wavelength at 490 nm and an emission wavelength of 530 nm. Serially diluted FITC-dextran in serum was.used to establish a standard curve.

### HPLC analysis

For HPLC analysis of lactulose and mannitol, urine samples were thawed, mixed thoroughly and centrifuged for 5 min at 20,000 × *g* and 4 °C. Fifty microliters of supernatant was mixed with 175 µl of HPLC-grade water and 25 µl of 20% sulfosalicylic acid fresh solution, followed by incubation for 15 min at room temperature. After storage at −20 °C overnight, diluted samples were thawed again, centrifuged for 10 min at 20,000 × *g* and 4 °C. An amount of 200 mg ion-exchange resins (TMD-8 hydrogen and hydroxide form, Sigma-Aldrich Chemie GmbH, Taufkirch, Germany) was added, mixed well and incubated for 30 min at room temperature. After centrifugation for 10 min at 20,000 × *g* and 4 °C, supernatants were subjected for HPLC analysis using a DIONEX UltiMate® 3000 system with Corona Veo SD Charged-Aerosol-Detector (Thermo Scientific GmbH, Dreieich, Germany). For chromatographic separation, a hydrophilic interaction liquid chromatography (HILIC) column (Acclaim™ Trinity™ P2, 3 µm, 3.0 × 100 mm, Thermo Scientific) was used. The eluent was a mixture of acetonitrile and 100 mM ammoniumformate (pH 3.65) with an isocratic ratio of 82:18 [vol/vol] at 0.6 ml/min and 60 °C column oven temperature. For determination of sucralose, urine samples were thawed, mixed thoroughly and centrifuged for 10 min at 20,000 × *g* and 4 °C. Fifty microliters of supernatant was mixed with 200 µl of HPLC-grade water, mixed thoroughly and centrifuged again for 10 min at 20,000 × *g* and 4 °C. Supernatants were analyzed by using a DIONEX UltiMate® 3000 system with Corona Veo SD Charged-Aerosol-Detector (Thermo Scientific GmbH, Dreieich, Germany). For chromatographic separation a Synergi 4µ Polar-RP 80A column was used (250 ×1.6 mm (Phenomenex, Aschaffenburg, Germany)). The eluent was a mixture of methanol and HPLC-grade water with an isocratic ratio of 30:70 [vol/vol] at 1.0 ml/min and 50 °C column oven temperature.

### Histological grading of intestinal inflammation in mice

Upon sacrifice, proximal duodenal, jejunal, ileal and colon tissue were harvested and fixed in 10% phosphate-buffered formalin. These samples were embedded in paraffin, sectioned at 4 μm, and stained with H&E or PAS for light microscopy examination. The slides were reviewed in a blinded fashion by a pathologist and were assigned a histological score for intestinal inflammation.

### Bone histology

Tibial bones and inflamed paws with tarsal joints were fixed in 4% formalin for 24 h and decalcified in Ethylenediaminetetraacetic acid (EDTA) (Sigma-Aldrich). Serial paraffin sections (2 μm) were stained for tartrate-resistant acid phosphatase (TRAP) using a Leukocyte Acid Phosphatase Kit (Sigma) according to the manufacturer’s instructions. Osteoclast numbers were quantified using a microscope (Carl Zeiss) equipped with a digital camera and an image analysis system for performing histomorphometry (Osteomeasure; OsteoMetrics).

### Immunofluorescence microscopy

Paraffin-embedded tissue sections were deparaffinized with xylene and rehydrated through graded alcohol solutions. Heat-induced epitope retrieval was performed by placing the sections in Retrieval Solution pH 9, diluted 1:20 (cat. EB-DEPP-9; eubio) at 85 °C for 20 min, washed three times with PBS and blocked with PBS containing 0.2% bovine serum albumin (BSA) and 0.1% saponin for 1 h at room temperature. The sections were then incubated with primary antibodies against occludin (Abcam, dilution 1:100) and ZO-1 (ThermoFisher, dilution 1:100), diluted in PBS containing 0.2% BSA and 0.1% saponin at 4 °C overnight, washed, and incubated for 1 h with Alexa Fluor 647 donkey anti-rabbit IgG secondary antibody (Invitrogen, dilution 1:400). Some sections were incubated with PBS containing 0.2% BSA and 0.1% saponin alone in place of primary antibody as a negative control. After having been washed three times with PBS, the sections were mounted and visualized. Imaging was performed by using the Leica DMi8 TIRF Widefield Fluorescence of ×10 objective lens (Leica, Germany) and Zeiss Spinning Disc Axio Observer Z1 of ×63 objective lens (Carl Zeiss) microscopes.

To compare ZO-1 and occludin levels, we considered three samples for each of the consecutive time points (day 0, 25, 30, 35, 40, 45 and 50) in ileum and colon for each of the proteins with three images from different parts of each section. Microscopy was performed and fluorescence images were visualized and captured in DAPI and Cy5 channels. Quantification of the images was performed using ImageJ software based on the mean fluorescence intensity. We calculated for each of the samples and condition the mean intensity across the entire image and divided it by the mean intensity of the DAPI channel to account for varying tissue sizes.

### Fecal supernatants preparation

Intestinal stool samples were obtained from healthy and CIA mice, at different time points of disease. Samples were suspended at 10% (wt/vol) in Dulbecco’s PBS, homogenized, and centrifuged at 4500 rpm for 10 min at 4 °C. Supernatants were passed through 1.2 µm membrane filters.

### Cell culture

Caco-2 cells, a human colorectal carcinoma-derived epithelial cell line, were cultivated in Dulbecco’s modified Eagle’s medium (DMEM)/F12 (Gibco) containing 10% fetal bovine serum (FBS) in a humidified incubator at 37 °C, 5% CO_2_. Cells were maintained for 14 days post confluency, serum-starved overnight and stimulated with FSN for 24 h or PBS as a control.

### Transepithelial resistance measurements

Caco-2 cells were seeded onto 12 mm polycarbonate membrane inserts (PCF membrane inserts, Millipore, Germany) and bathed in DMEM/F12 medium which contained phenol red, 10% FBS, penicillin (100 units/ml) and streptomycin (10 µg/ml). Cells were continuously maintained in a humidified incubator at 37 °C, 5% CO_2_. To assess that these cells form monolayer on the membrane inserts, transepithelial resistance was routinely monitored using an epithelial volt-ohm-meter including STX-2 electrodes (EVOM, World Precision Instruments, Berlin, Germany). After 12–16 days post seeding, membrane inserts containing confluent monolayer of Caco-2 cells were transferred into Ussing chambers^47,48^. The basolateral bath contained 1.0 ml and the apical bath 0.5 ml of the supplemented DMEM/F12 medium. The transepithelial resistance was recorded every 10 s using a CVC6 clamp device (Fiebig, Berlin Germany)^49^ by measuring voltage deflections induced by injection of 20 µA symmetrical square pulses for 0.4 s. After an equilibration period of 10–40 min, AT-1002 (5 mg/ml) was applied to the apical bath by replacing 400 µl of the bath medium with 400 µl of medium containing the AT-1002 component. In time matched controls the dissolvent of AT-1002 was added to the apical bath. The transepithelial resistance was then continuously recorded for a further 3 h.

### Flow cytometry

Small intestines (all data shown for the ileum) and colons were analyzed by flow cytometry. In short, tissues were incubated with PBS containing EDTA at 37 °C for 2 × 20 min, then digested twice for 35 min with a cocktail of collagenase D (1 mg/ml, Roche), dispase I (0.1 mg/ml, Roche), and DNase I (333 mg/ml, Sigma), smashed and filtered through a 40 mm gauze (BD Biosciences). Single-cell suspensions were then stimulated with phorbol 12-myristate 13 acetate and ionomycin (Sigma-Aldrich) for 6 h; monensin (BD-Bioscience) and brefeldin A (BioLegend) were added after 2 h or directly stained with the respective directly labeled FACS antibodies. All intracellular stainings were used in combination with a FoxP3staining kit (eBioscience). Flow cytometric analysis was performed on a Gallios Flow Cytometer (Beckman Coulter) or CytoFLEX (Beckmann Coulter) and evaluated using the Kaluza and cytExpert Flow Cytometry analysis software (Beckman Coulter). Antibodies used: Alexa Fluor® 647 anti-mouse CD3 antibody (BioLegend, 1:600); PE/Cy7 anti-mouse CD4 antibody (BioLegend, 1:400); Alexa Fluor® 700 anti-mouse CD8a antibody (BioLegend, 1:600); PE Mouse anti-Mouse RORγt (BD Pharmingen, 1:100); Pacific Blue™ anti-T-bet Antibody (BioLegend, 1:100); Alexa Fluor® 647 anti-mouse FOXP3 Antibody (BioLegend, 1:100); PE anti-GATA3 Antibody (BioLegend, 1:100); Brilliant Violet 421™ anti-mouse CD3ε Antibody (BioLegend, 1:200); APC/Cy7 anti-mouse/human CD45R/B220 Antibody (BioLegend, 1:200); Alexa Fluor 700 anti-mouse/human CD11b Antibody (BioLegend, 1:200); CD11c Monoclonal Antibody (N418), APC (eBioscience™, 1:400); APC anti-mouse IFN-Antibody (BioLegend, 1:50); BV421 Rat Anti-Mouse IL-4 (BD Biosciences, 1:200); PE anti-mouse IL17A Antibody (BioLegend, 1:200).

### Intestinal organoid isolation and cultivation

The small intestine of wild-type C57Bl/6J was removed and washed with ice-cold PBS. It was opened longitudinally and villi were scraped out using a coverslip and discarded. The intestine was cut into 2–3 mm pieces and collected in ice-cold PBS. After sedimentation the supernatant was discarded and fresh ice-cold PBS was added to resuspend the intestinal pieces. This procedure was repeated 15–20 times until the supernatant was clear. The intestinal pieces were then incubated at 4 °C for 20–30 min in ice-cold PBS containing 2 mM EDTA (Merck) while agitating. Afterwards the tissue was sedimented by gravity and the supernatant was replaced by ice-cold PBS. The intestinal crypts were dissociated from the tissue by vigorous shaking. The supernatant was collected and screened for crypts by microscopy. This step was repeated until no crypts were left in the supernatant. The supernatant fractions enriched with crypts were passed through a 70 µm cell strainer and centrifuged at 300 × *g* for 5 min at 4 °C. The pelleted crypts were resuspended in 25 µl/well growth factor reduced Matrigel (Corning, New York, USA). The cell suspension was then distributed into a 48-well cell culture plate. The Matrigel was allowed to polymerize in the incubator at 37 °C and 5% CO_2_ for 20 min. Afterwards 300 µl of Intestinal Organoid Growth medium (mouse) (Stemcell, Köln, Germany) was added per well. Organoids were cultured in the incubator for at least 7 days before any experiments were performed. Medium was changed every 2–3 days and organoids were splitted once per week^50^.

### Permeability assay

For imaging organoids were plated in eight-well chamber slides (Ibidi, Planegg, Germany). The organoids were imaged using a Zeiss Spinning Disc confocal microscope (Zeiss, Jena, Germany). At the beginning of the experiment 1 mM Lucifer yellow (VWR International GmbH, Darmstadt, Germany) was added to each of the chambers. Afterwards the organoids were imaged for 100 min with intervals of 5 min. To ensure the capability of the organoids to get permeable, 1 mM of EGTA (Merck) was added after the observation time. Following EGTA addition organoids were imaged for another 20 min. For each time point lucifer yellow fluorescence inside and outside the organoids was quantified using Fiji ImageJ. Only organoids that were able to take up Lucifer yellow after EGTA treatment were analyzed^51^.

### Western blotting

Caco-2 cells were washed twice with PBS and subsequently lysed. Intestinal tissue samples were homogenized in protein lysis buffer and subsequently sonicated. Protein extracts were separated on 8% SDS-polyacrylamide or 4–12% bis−tris protein gel (Invitrogen), transferred on a nitrocellulose membrane, and stained with antibodies against ZO-1 (N1N2, GeneTex, dilution 1:3000). An antibody against β-actin (AC-15, abcam, dilution 1:25,000) was used as loading control. Supplementary Fig. 10 shows the uncropped gel scans of Western blots.

### SCFA measurements

Five replicates of frozen cecal samples (100 mg) were weighed into a 2 ml polypropylene tube. The tubes were kept in a cool rack throughout the extraction. 33% HCl (50 µl for cecal contents or 5 µl for serum) was added and samples were vortexed for 1 min. One milliliter of diethyl ether was added, vortexed for 1 min and centrifuged for 3 min at 4 °C. The organic phase was transferred into a 2 ml gas chromatography (GC) vial. For the calibration curve, 100 μl of SCFA calibration standards (Sigma) were dissolved in water to concentrations of 0, 0.5, 1, 5 and 10 mM and then subjected to the same extraction procedure as the samples. For GCMS analysis 1 μl of the sample (4–5 replicates) was injected with a split ratio of 20:1 on a Famewax, 30 m x 0.25 mm iD, 0.25 μm df capillary column (Restek, Bad Homburg). The GC-MS system consisted of GCMS QP2010Plus gas chromatograph/mass spectrometer coupled with an AOC20S autosampler and an AOC20i auto injector (Shimadzu, Kyoto, Japan). Injection temperature was 240 °C with the interface set at 230 °C and the ion source at 200 °C. Helium was used as carrier gas with constant flow rate of 1 ml/min. The column temperature program started with 40 °C and was ramped to 150 °C at a rate of 7 °C/min and then to 230 °C at a rate of 9 °C/min, and finally held at 230 °C for 9 min. The total run time was 40 min. SCFA were identified based on the retention time of standard compounds and with the assistance of the NIST 08 mass spectral library. Full scan mass spectra were recorded in the 25–150 m/z range (0.5 s/scan). Quantification was done by integration of the extracted ion chromatogram peaks for the following ion species: m/z 45 for acetate eluted at 7.8 min, m/z 74 for propionate eluted at 9.6 min, and m/z 60 for butyrate eluted at 11.5 min. GCMS solution software was used for data processing.

### Micro-computed tomography

µCT imaging was performed using the cone-beam Desktop Micro Computer Tomograph “µCT 40” by SCANCO Medical AG, Bruettisellen, Switzerland. The settings were optimized for calcified tissue visualization at 55 kVp with a current of 145 µA and 200 ms integration time for 500 projections/180°. For the segmentation of 3D-volumes, an isotropic voxel size of 8.4 µm and an evaluation script with adjusted grayscale thresholds of the operating system “Open VMS” by SCANCO Medical was used. Volume of interest tibia: The analysis of the bone structure was performed in the proximal metaphysis of the tibia, starting 0.43 mm from an anatomic landmark in the growth plate and extending 1.720 mm (200 tomograms) distally.

### Specific IgG detection via ELISA

Collagen type II (CII)-specific antibodies were detected by ELISA using high-binding plates (Nunc) coated overnight with 10 μg/ml mouse or chicken CII. The detection of mouse CII-specific antibodies was carried out by Eu3+-labeled anti-mouse IgG antibody and the DELFIA system (PerkinElmer) according to the manufacturer’s recommendations.

### 16S rRNA microbiome analysis

For Supplementary Fig. 4a–d, genomic DNA was extracted according to Qiamp Fast DNA Stool extraction kit (Qiagen) and the V3–V4 region of the bacterial 16S rRNA gene was amplified with the NEBNext Q5 Hot Start Hifi PCR Master Mix (NEB) using a dual-index strategy. Amplified fragments were purified with AMPure XP Beads (Beckmann Coulter Genomics), combined and analyzed by “2 × 300 bp paired-end” sequencing using an Illumina MiSeq device. Quality control, OTU table generation and bioinformatics analysis was done using the Usearch10 (Edgar RC Flyvbjerg H, 2015) and the Microbiomeanalyst package^52^. The database of the “Ribosomal” database project (RDP release 16) was used for classification. For Supplementary Fig. 4e, microbial DNA extraction from fecal content was done using ZymoBIOMICS 96 Magbead DNA Kit automated on a Tecan Fluent 480 liquid handling system. Amplification of the V4 region (F515/R806) of the 16S rRNA gene was performed as in previously described protocols^53^. Samples were sequenced on an Illumina MiSeq platform (PE250). Barcode-based demultiplexing was performed using IDEMP software with default parameters (https://github.com/yhwu/idemp). Obtained reads were assembled, quality controlled, and clustered using Usearch8.1 software package (http://www.drive5.com/usearch/). Briefly, reads were merged using -fastq_mergepairs –with fastq_maxdiffs 30 and quality filtering was done with fastq_filter (-fastq_maxee 1), minimum read length 200 bp. The OTU clusters and representative sequences were determined using the UPARSE algorithm^54^, followed by taxonomy assignment using the Silva database v128^55^ and the RDP Classifier^56^ with a bootstrap confidence cutoff of 80% performed by using QIIME v1.8.0^57^. OTU absolute abundance table and mapping file were used for statistical analyses and data visualization in the R statistical programming environment package phyloseq^58^ or web-based tool MicrobiomeAnalyst^52^.

### Statistical analysis

Data are expressed as mean ± s.d. unless otherwise indicated in the figure legends. Analysis was performed using Student’s *t* test, single comparison, or analysis of variance (ANOVA) test for multiple comparisons (one-way or two-way ANOVA followed by Tukey’s or Bonferroni’s multiple comparisons test, respectively). All experiments were conducted at least two times. *P* values of 0.05 were considered significant and are shown as *p < 0.05, **p < 0.01, or ***p < 0.001. Graph generation and statistical analyses were performed using the Prism version 8 software (GraphPad, La Jolla, CA).

## Supplementary information

Supplementary Information

## Data Availability

All relevant data are available from the authors upon reasonable request. The source data underlying Figs. 1–6 and Supplementary Figs. 1–10 are provided as a Source data file. Sequence data are deposited in BioProject with the accession code PRJNA592061.

## Source data

Source Data
