# Supplementary material for: Targeting zonulin and intestinal epithelial barrier function to prevent onset of arthritis
Source: Nat Commun. 2020 Apr 24;11:1995. doi: 10.1038/s41467-020-15831-7 (PMC7181728; doi:10.1038/s41467-020-15831-7)
Supplement: Supplementary file 1 — Supplementary Information [file 41467_2020_15831_MOESM1_ESM.pdf]

Targeting zonulin and intestinal epithelial barrier function to prevent onset of Arthritis

Tajik et al.

Supplementary Information

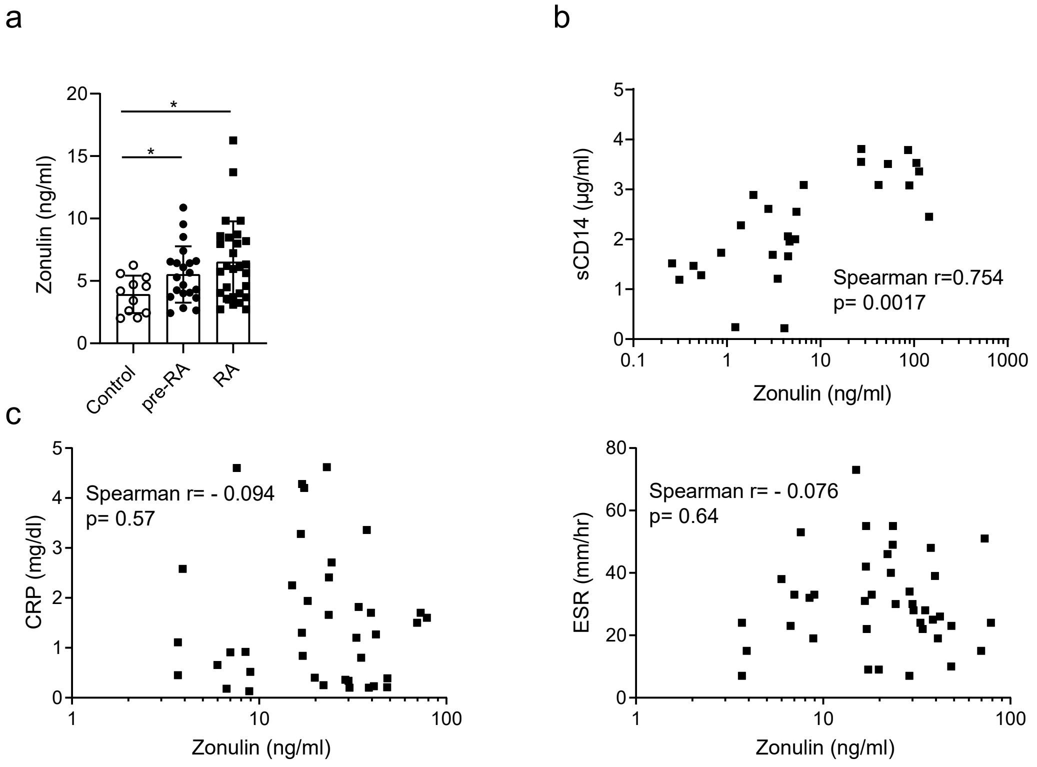

Supplementary Figure 1. Serum zonulin in pre-RA patients positively correlate with serum sCD14 concentrations from same patients. (a) Serum zonulin levels in healthy control (control) (n=11), pre-rheumatoid arthritis (RA) patients (n=21) and RA patients (=28) from a cohort from Chengdu, China. (b) Serum from pre-RA patients shown in Fig.1b for zonulin concentrations were additionally analyzed for sCD14 concentrations. (c) Serum from pre-RA patients shown in Fig. 1b for zonulin concentrations were additionally analyzed for CRP and ESR concentrations. Data are expressed as mean ± s.d. Statistical difference was determined by one-way ANOVA and Spearman r correlation. *= p<0.05. Source data are provided as a Source Data file.

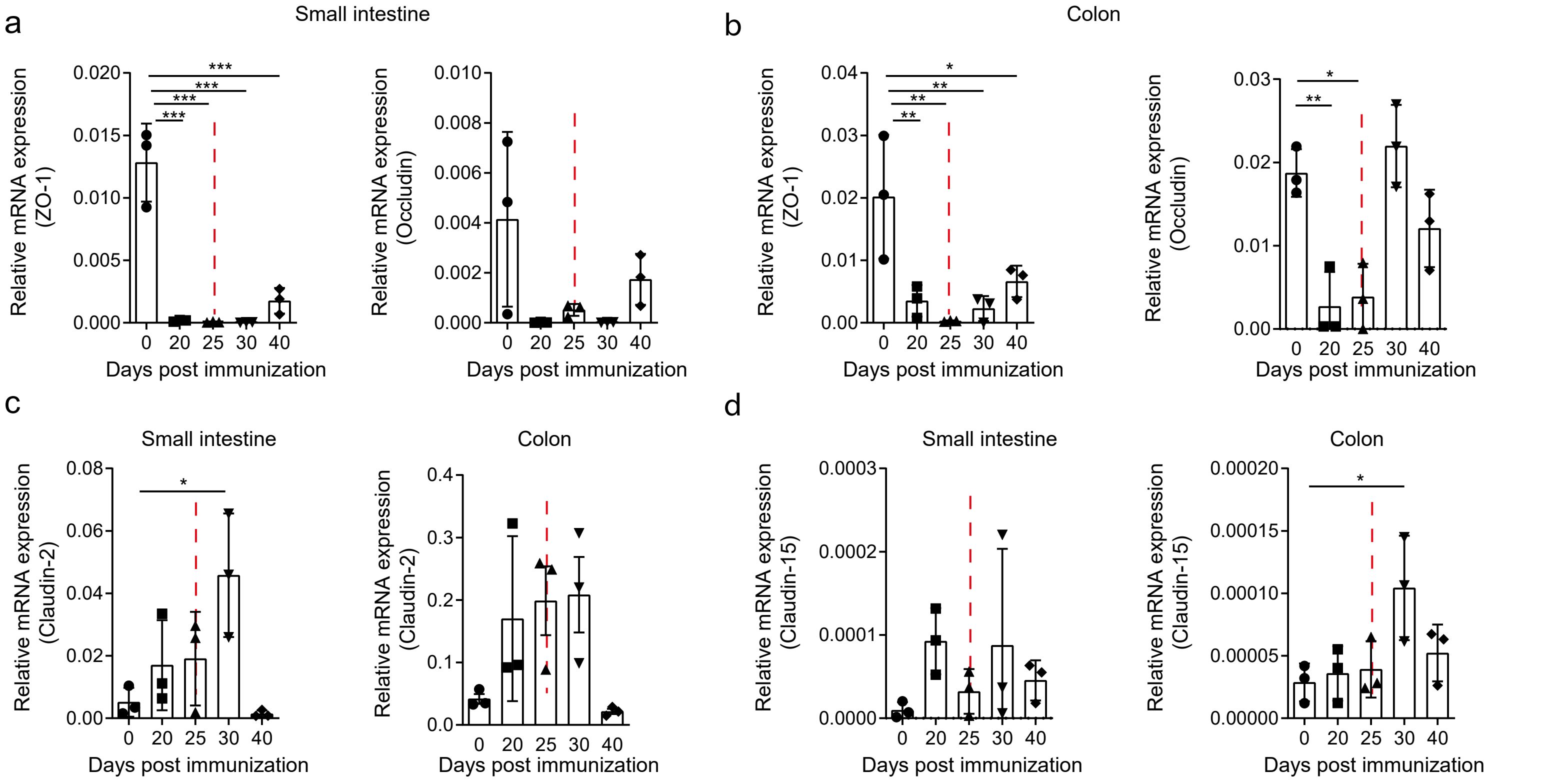

Supplementary Figure 2. Tight junction mRNA expression levels in CIA mice.

(a) relative mRNA expression of ZO-1 and occludin in collagen-induced arthritis (CIA) mice in ileal tissue samples. (b) Relative mRNA expression of ZO-1 and occludin in CIA mice in colon tissue samples. (c) Relative mRNA expression of claudin-2 in CIA mice in ileal and colon tissue samples. (d) Relative mRNA expression of claudin-15 in CIA mice in ileal and colon tissue samples. Data are derived from 2 independent experiments with n=3 and expressed as mean ± s.d. Statistical difference was determined by one-way ANOVA. *= p<0.05; **= p<0.01; ***= p<0.001. Source data are provided as a Source Data file.

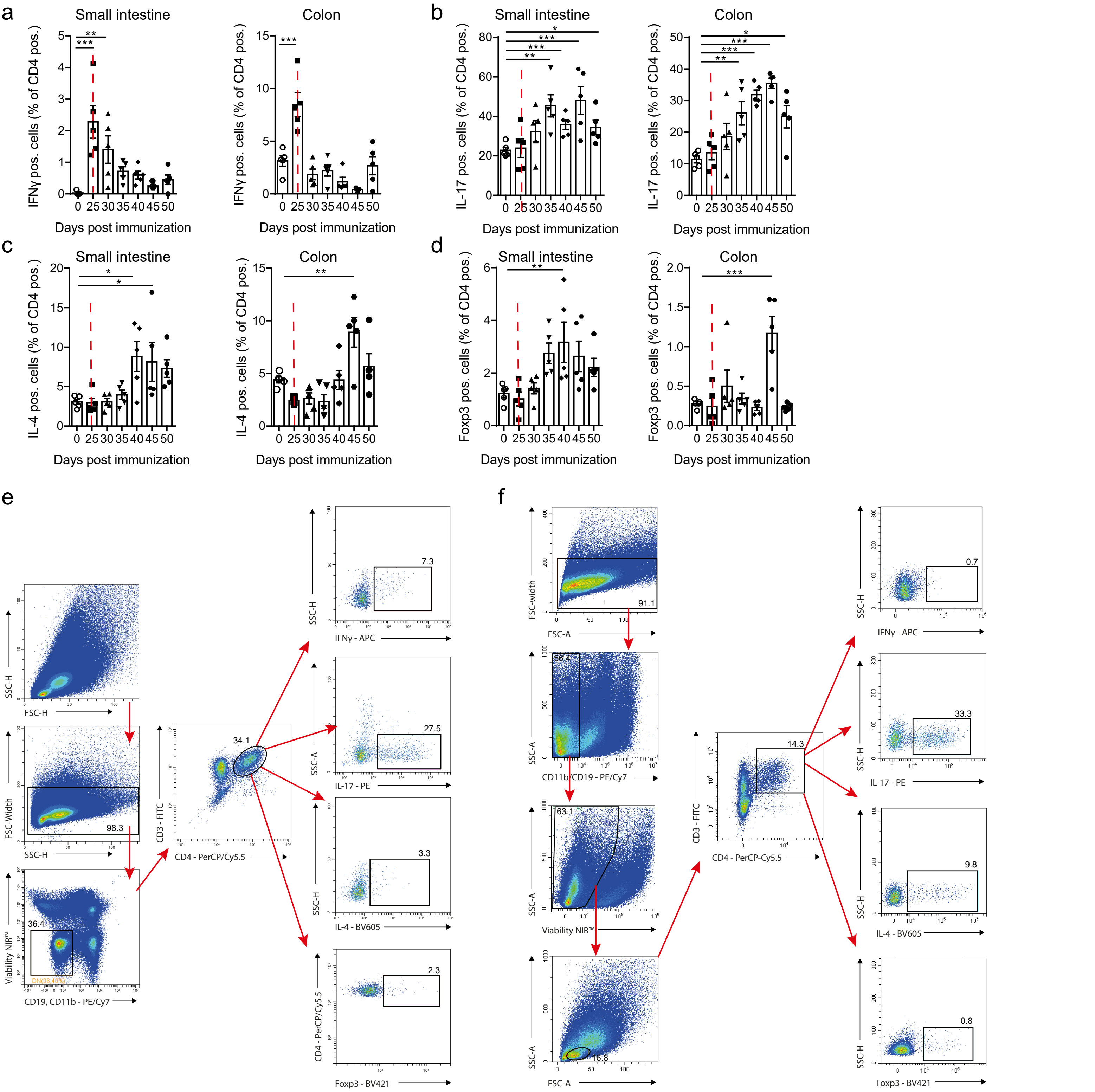

Supplementary Figure 3. Flow cytometry analyis of T cell substes in the intestine of CIA mice.
Flow cytometry analysis of lamina propria (a) IFNγ+, (b) IL-17+, (c) IL-4+, and (d) Foxp3+ CD4+ T cells from small intestinal and colon tissue of collagen-induced arthritis (CIA) mice. (e) Flow cytometry-gating strategy shown from ileal samples exemplarily from day 25 post immunization in collagen-induced arthritis (CIA) DBA/1 mice. (f) Flow cytometry-gating strategy shown from colon samples exemplarily from day 45 post immunization in collagen-induced arthritis (CIA) DBA/1 mice. Data are derived from 3 independent experiments with n=5 and expressed as mean ± s.d. Statistical difference was determined by one-way ANOVA. *= p<0.05; **= p<0.01; ***= p<0.001. Source data are provided as a Source Data file.

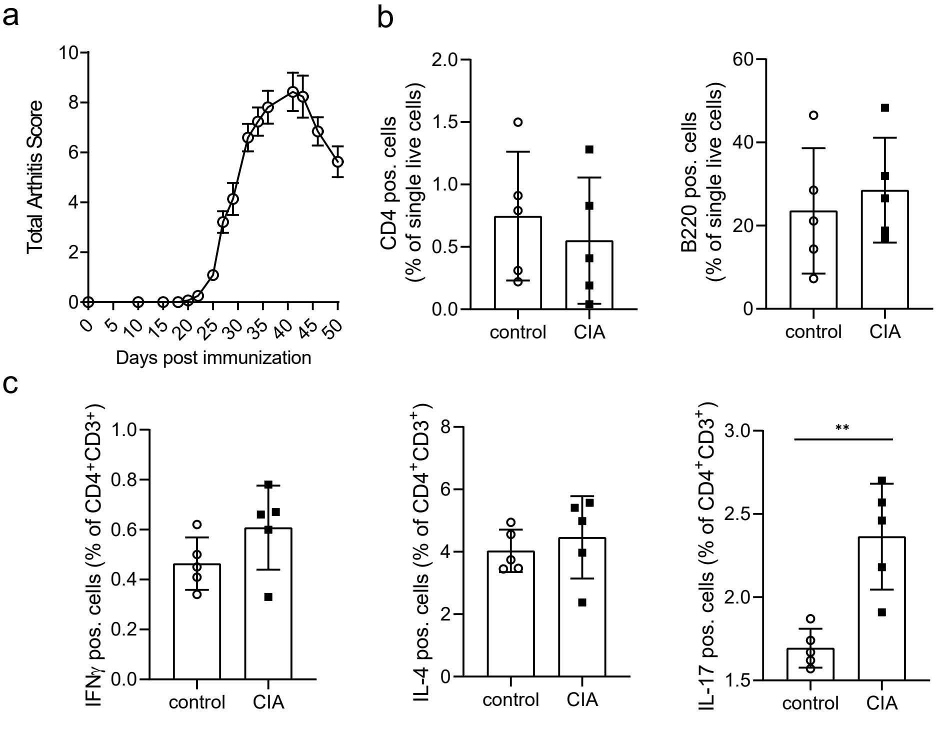

Supplementary Figure 4. Clinical arthritis scores and flow cytometry analysis of synovial tissue and popliteal lymph nodes (pLN) of CIA mice. Time course and total arthritis scores in collagen-induced arthritis (CIA) mice (n=16). (b) Synovial tissue cells detected at 25 days post immunization (dpi) (n=5). (c) pLN cells detected at 25 dpi (n=5). Data are derived from xx independent experiments and expressed as mean ± s.d. Statistical difference was determined by one-way ANOVA. *= p<0.05; **= p<0.01; ***= p<0.001. Source data are provided as a Source Data file.

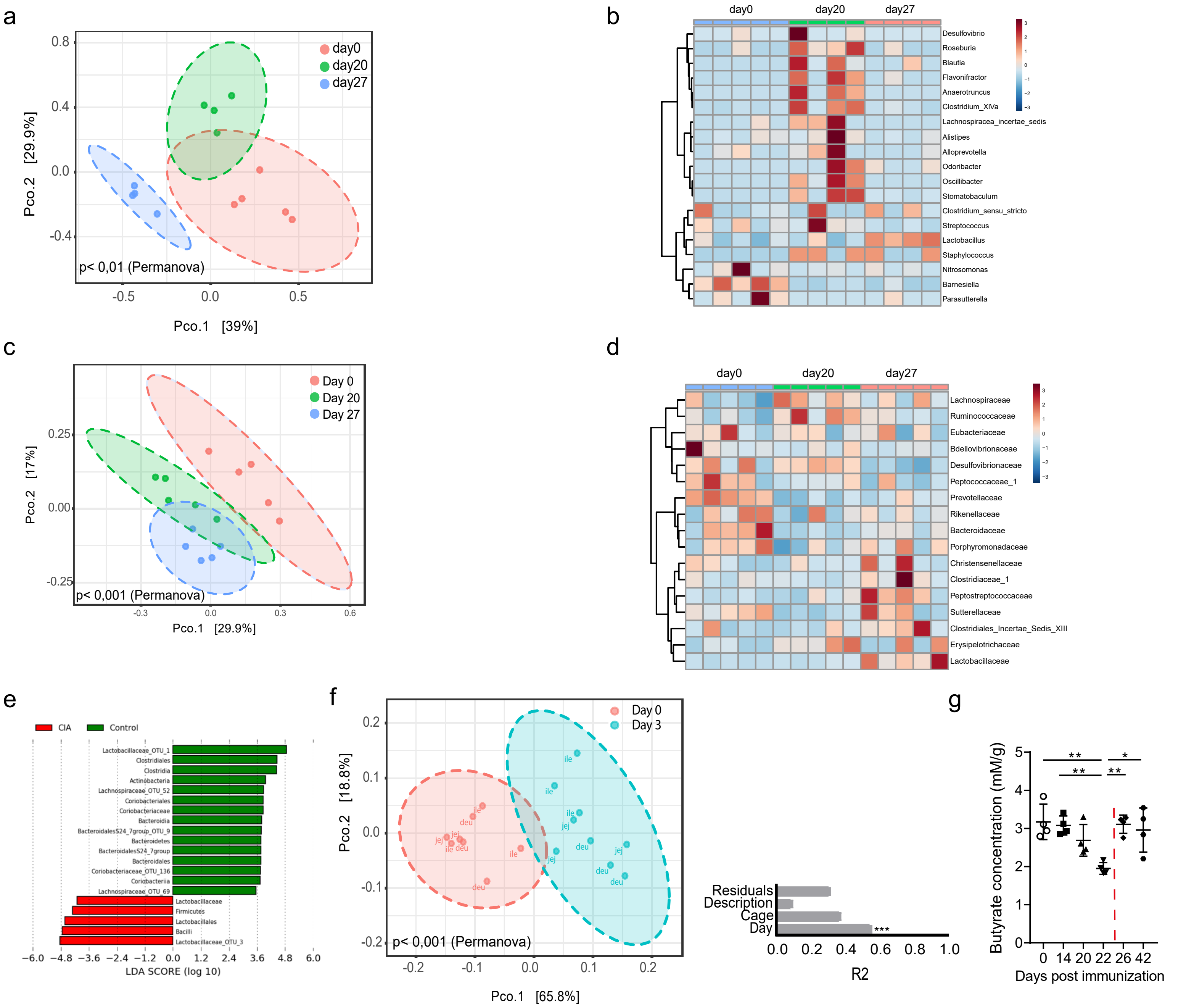

Supplementary Figure 5. Reduced cecal butyrate levels and intestinal microbial dysbiosis during the early initiation phase in CIA mice.
(a) Principal coordinate analysis (PCoA) of pairwise bray curtis dissimilarities between ileal microbiota samples (OTU level).
(b) Heat map representation of relative abundances of different bacterial genera in ileal stool samples.
(c) Principal coordinate analysis (PCoA) of pairwise bray curtis dissimilarities between cecal microbiota samples (OTU level).
(d) Heat map representation of relative abundances of different bacterial families in cecal stool samples. (e) LDA scores representing taxa with relative abundance > 0.5% in the ileum of CIA mice at day 0 vs. day 3 post immunisation. (f) Principal coordinate analysis (PCoA) of whole small intestine at day 0 and day 3 post immunisation in CIA mice alongside with multivariate analysis of variance (ADONIS). (g) Cecal butyrate concentrations during CIA time course (n=4). Data are derived from 2 independent experiments with n=3 and expressed as mean ± s.d. Statistical difference was determined by one-way ANOVA. *= p<0.05; **= p<0.01; ***= p<0.001. Source data are provided as a Source Data file and found under following accession code, BioProject ID: PRJNA592061.

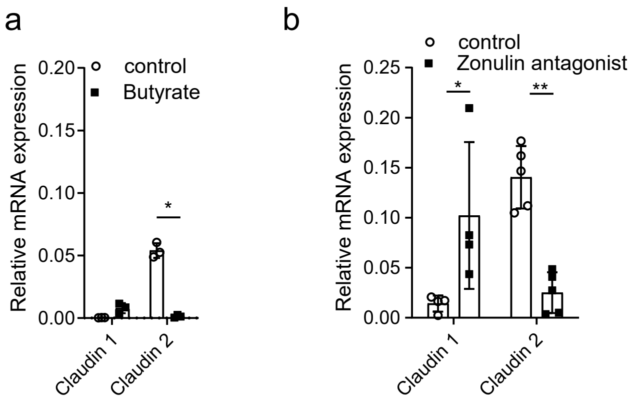

Supplementary Figure 6. Claudin expression mRNA levels.
(a) RT-PCR analysis of mRNA expression of claudins in intestinal organoid cultures stimulated with butyrate (n=3).
(b) Claudin mRNA expression profiles in ileal tissue samples in collagen-induced arthritis mice treated with zonulin antagonist (n=5). Data are derived from 2 independent experiments and expressed as mean ± s.d. Statistical difference was determined by one-way ANOVA. *= p<0.05; **= p<0.01; ***= p<0.001. Source data are provided as a Source Data file.

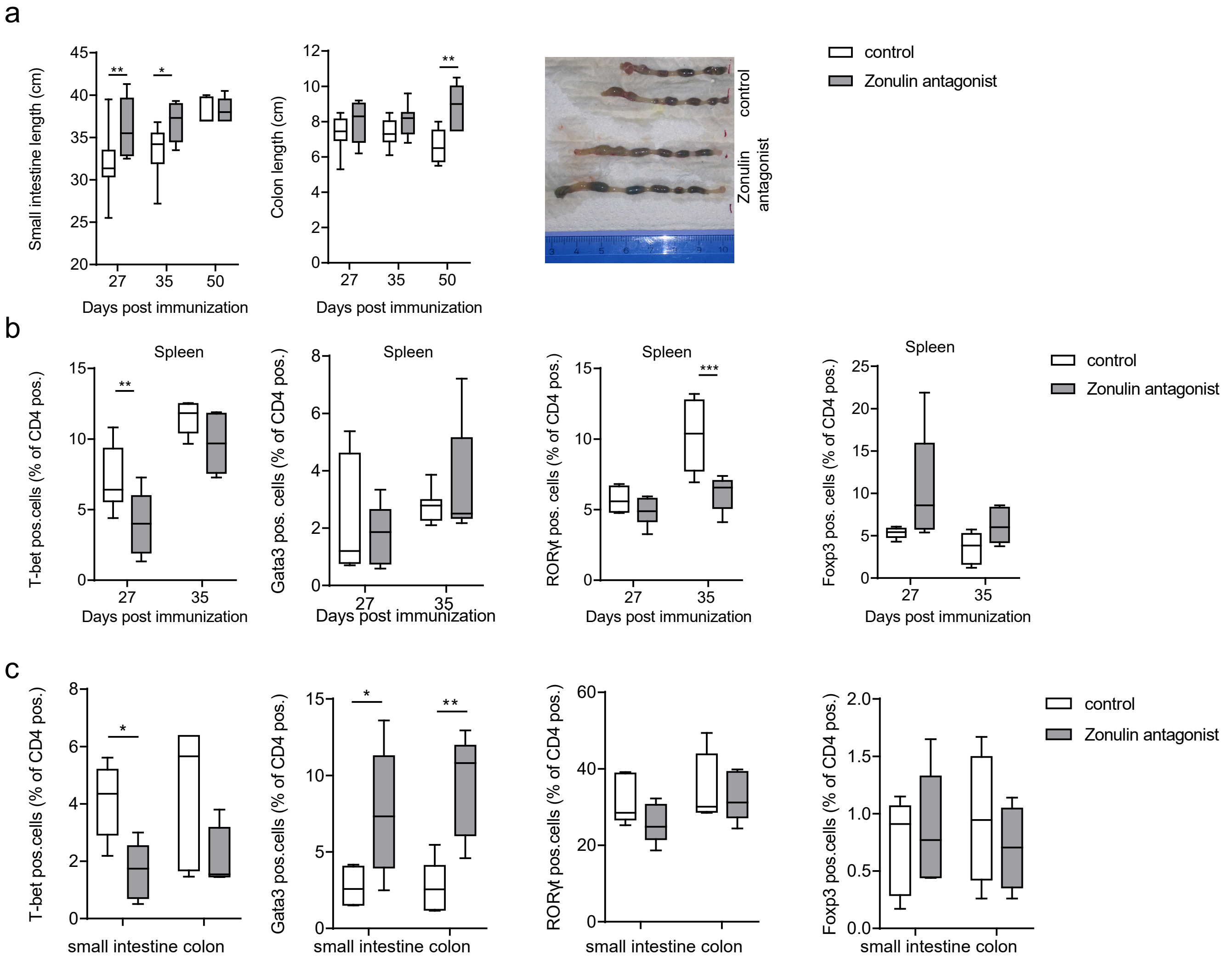

Supplementary Figure 7. Zonulin-inhibition increases Tregs and prevents cell trafficking from the intestine to the joints.
(a) Length of small intestine and colon in mice induced for CIA and treated with zonulin antagonist.
(b) Splenic T-bet+, Gata3+, RORγt+, and Foxp3+ CD4+ T cells after zonulin antagonist treatment.
(c) T-bet+, Gata3+, RORγt+, and Foxp3+ CD4+ T cells in intestines after zonulin antagonist treatment 27 days post immunization.
Data are derived from 3 independent experiments with n=5 presented as box and whiskers showing 1-99 percentile. Statistical difference was determined by two-way ANOVA. p*= $p<0.05$; p**= $p<0.01$; p***= $p<0.001$.Source data are provided as a Source Data file.

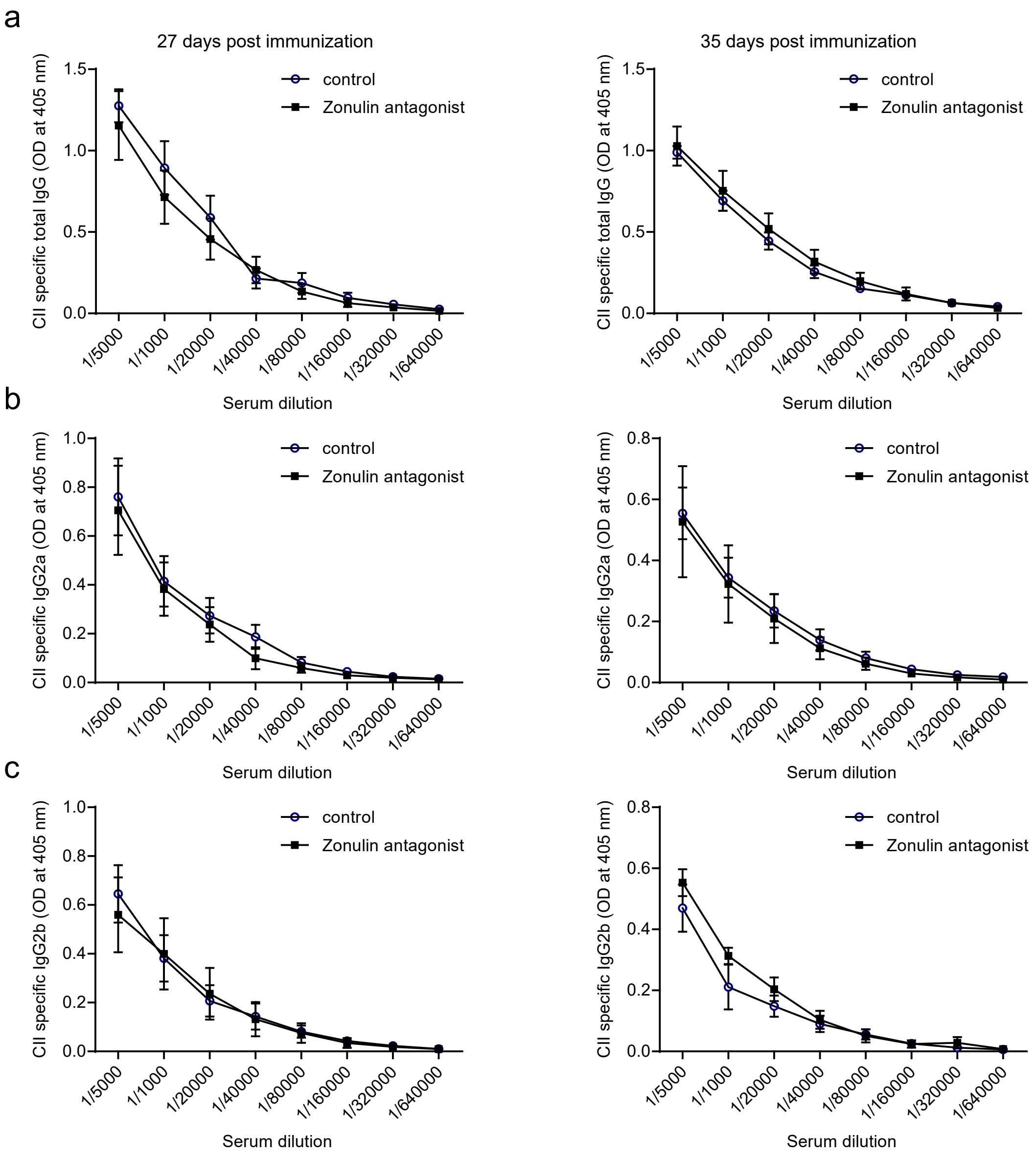

Supplementary Figure 8. No effects of zonulin antagonist treatment on serum CII-specific Ig titres.
(a)CII-specific total IgG during collagen-induced arthritis (CIA) at 27 days post immunization (dpi) and 35 dpi with or without zonulin antagonist treatment. (b) CII-specific IgG2a during CIA at 27 days post immunization (dpi) and 35 dpi with or without zonulin antagonist treatment. (c) CII-specific IgG2b during CIA at 27 days post immunization (dpi) and 35 dpi with or without zonulin antagonist treatment. Data are derived from 3 independent experiments with n=5 and expressed as the mean ± s.d.. Source data are provided as a Source Data file.

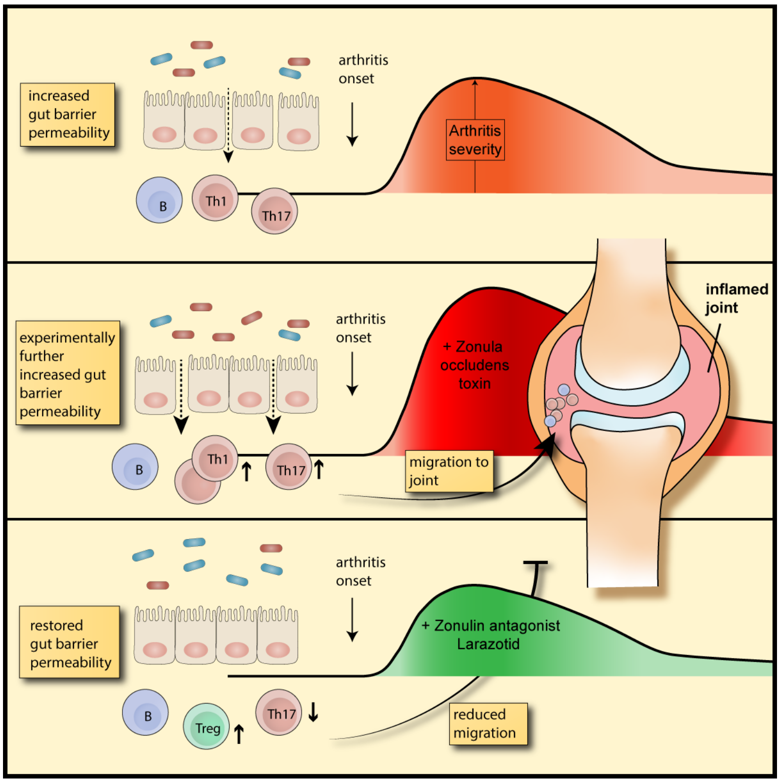

Supplementary Figure 9. Graphical summary of key findings.

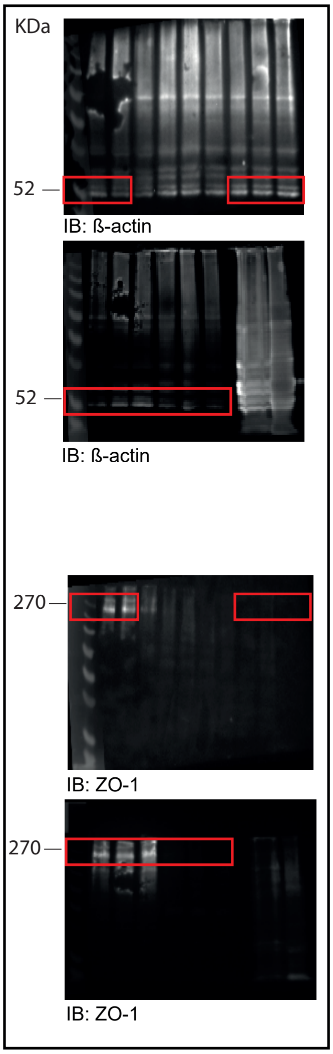

Supplementary Figure 10. Uncropped original Western Blot scans shown in Figure 3e.

	Age (Mean± SD)	Age (median, IQR)	Sex (%females)	BMI (Mean± SD)	BMI (median, IQR)	DAS28 (Mean± SD)	DAS28 (median,IQR)
**Cohort 1 (Erlangen)**							
Healthy Controls (N=41)	49.3 ± 10.2	50 (42;54)	100%	27.8 ± 3.3	28 (25;30)	-	-
Pre-RA (N=65)	51.1 ± 10.2	50 (44;61)	100%	27.5 ± 2.9	27 (26;29)	-	-
Pre-RA progressors (N=12)	51.0 ± 10.4	50 (47,5;60,5)		27.0 ± 1.8	27 (26;27,75)	-	-
Pre-RA –non progressors (N=53)	51.1 ± 10.3	49 (44;61)		27.6 ± 3.1	27 (26;29)	-	-
RA (N=31)	52.7 ± 10.7	55 (46;61,75)	100%	28.0 ± 3.9	28 (25;30)	3.2 ± 0.8	2.9 (2.6;3.6)
	P=0.39			P=0.75			
**Cohort 2 (Palermo)**							
Healthy Controls (N=10)	46.5 ± 7.3	48 (41; 52)	7 (70)	30.3 ± 3.4	28 (26;33)	-	-
New-onset RA (N=10)	52.8 ± 6.3	54 (44;60)	80%	29.2 ± 2.6	30 (25;32)	3.1 ± 0.35	3.3 (2.6;3.5)
Established RA (N=5)	55.6 ± 8.8	57 (49; 66)	7 (70)	31.3 ± 3.2	31 (26; 33)	4.4 ± 1.2	4 (3.5; 4.6)
	P=0.44		P=0.33	P=0.44			
**Cohort 3 (Chengdu)**							
Healthy Controls (N=11)	44.8 ± 8.7	47 (42;50)	100%	24.0 ± 2.3	25 (22;26))	-	-
Pre-RA (N=21)	49.2 ± 6.8	51 (47;51)	100%	23.5 ± 3.0	24 (22;26)	-	-
RA (N=28)	48.1 ± 11.2	50 (43;55)	100%	24.5 ± 2.0	24 (22;25)	4.6 ± 1.6	4.4 (3.4;4.9)
	P=0.27			P=0.65			
**Other Diseases**							
Glioblastoma (N=8)	59.9 ± 15.89	62 (46;72)	100%	29.21 ± 4.51	28.69 (25.4;31.3)	-	-
Metastatic Cancer (N=11)	59.58 ± 10.86	60.5 (49;66.75)	100%	22.31 ±3.76	20.87 (19.4;25.28)	-	-
Hepatitis C (N=21)	52.16 ± 10.09	54 (46, 61)	28%	-	-	-	-

Supplementary Table 1. Demographic characteristics of various patients and controls used for serum zonulin and serum sCD14 measurements. P values are based on ANOVA comparing the various groups. RA, rheumatoid arthritis; IQR, inter-quartile range, BMI, body mass index; DAS28, disease activity score 28.
